# Pinnisterols D–J, New 11-Acetoxy-9,11-secosterols with a 1,4-Quinone Moiety from Formosan Gorgonian Coral *Pinnigorgia* sp. (Gorgoniidae)

**DOI:** 10.3390/md15010011

**Published:** 2017-01-06

**Authors:** Yu-Chia Chang, Tsong-Long Hwang, Liang-Mou Kuo, Ping-Jyun Sung

**Affiliations:** 1 National Museum of Marine Biology & Aquarium, Pingtung 944, Taiwan; jay0404@gmail.com; 2 Doctoral Degree Program in Marine Biotechnology, National Sun Yat-sen University and Academia Sinica, Kaohsiung 804, Taiwan; 3 Graduate Institute of Natural Products, College of Medicine, Chang Gung University, Taoyuan 333, Taiwan; htl@mail.cgu.edu.tw; 4 Research Center for Industry of Human Ecology, Research Center for Chinese Herbal Medicine, and Graduate Institute of Healthy Industry Technology, College of Human Ecology, Chang Gung University of Science and Technology, Taoyuan 333, Taiwan; 5 Department of Anesthesiology, Chang Gung Memorial Hospital, Taoyuan 333, Taiwan; 6 Graduate Institute of Clinical Medical Sciences, College of Medicine, Chang Gung University, Taoyuan 333, Taiwan; kuo33410@yahoo.com.tw; 7 Division of General Surgery, Department of Surgery, Chang Gung Memorial Hospital, Chiayi 613, Taiwan; 8 Graduate Institute of Marine Biology, National Dong Hwa University, Pingtung 944, Taiwan; 9 Chinese Medicine Research and Development Center, China Medical University Hospital, Taichung 404, Taiwan; 10 Department of Marine Biotechnology and Resources, National Sun Yat-sen University, Kaohsiung 804, Taiwan; 11 Graduate Institute of Natural Products, Kaohsiung Medical University, Kaohsiung 807, Taiwan

**Keywords:** 9,11-secosterol, gorgonian, *Pinnigorgia*, cytotoxicity, hepatic stellate cell line (HSC-T6), anti-inflammatory, superoxide anion, elastase

## Abstract

Seven new marine 11-acetoxy-9,11-secosterols, pinnisterols D–J (**1**–**7**), with a 1,4-quinone moiety, were discovered from the gorgonian coral *Pinnigorgia* sp. In this study, the structures of secosterols **1**–**7** were revealed by spectroscopic analysis. Bioactivity study showed that secosterol **1** treatment inhibited cell viability in a hepatic stellate cell line, HSC-T6, with an IC_50_ value of 3.93 μM; and secosterols **2**, **5**, and **7** reduced elastase enzyme release, and **3**, **5**, and **7** decreased the production of superoxide anions from human neutrophils.

## 1. Introduction

**Figure 1:**
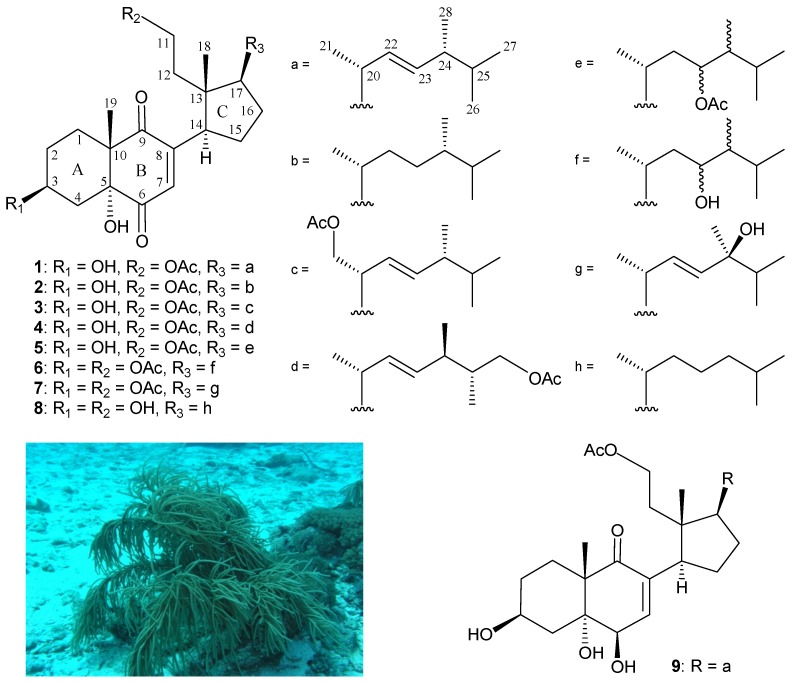
Chemical structures of pinnisterols D–J (**1**–**7**), sterol **8**, and pinnisterol A (**9**), and an image of gorgonian coral *Pinnigorgia* sp.

Since the isolation in 1972 of the first marine 9,11-secosteroid, 9-oxo-9,11-secogorgost-5-ene-3*β*, 11-diol 11-acetate, from the gorgonian coral *Pseudopterogorgia americana* [1], a series of compounds of this group has been prepared from various marine invertebrates, particularly sponges and octocorals, with complex structures and interesting bioactivities [2]. Our continued investigations of gorgonian coral *Pinnigorgia* sp. (phylum Cnidaria, class Anthozoa, subclass Octocorallia, order Alcyonacea, family Gorgoniidae) have yielded various interesting secondary metabolites [3,4,5,6,7,8], including 9,11-secosterols [3,4,5], some of which have been shown to possess bioactivity, such as anti-inflammatory and cytotoxic properties [3,4,5,6,7,8]. With the aim of discovering bioactive marine metabolites for new drug development in the future, we carried out an investigation of the chemical composition of the gorgonian coral *Pinnigorgia* sp. In this study, we performed compound preparation and structure determination, and investigated the cytotoxicity and anti-inflammatory activities of seven new 9,11-secosterols, pinnisterols D–J (**1**–**7**), following further study of *Pinnigorgia* sp. (Figure 1).

## 2. Results and Discussion

A new metabolite was isolated as a colorless oil, and was named pinnisterol D (**1**). The high-resolution electrospray ionization mass spectrum (HRESIMS) showed a signal at *m/z* 525.31853 (calcd. for C_30_H_4__6_O_6_ + Na, 525.31921), and therefore the molecular formula of **1** was determined to be C_30_H_4__6_O_6_ (8° of unsaturation). ^13^C and distortionless enhancement polarization transfer (DEPT) experimental data indicated that **1** had 30 carbons, including seven methyls, seven sp^3^ methylenes (including one oxymethylene), six sp^3^ methines (including one oxymethine), two sp^3^ quaternary carbons, one sp^3^ tertiary alcohol, three sp^2^ methines, one sp^2^ tertiary carbon, two ketonic carbonyls, and one ester carbonyl (Table 1). In addition, IR spectroscopy demonstrated that the compound contained hydroxy (ν_max_ 3446 cm^−1^), ester (ν_max_ 1741 cm^−1^), and *α*,*β*-unsaturated ketonic carbonyl (ν_max_ 1685 cm^−1^) groups. The latter structural feature of **1** was further proven by the presence of signals at δ_C_ 197.5 (C-6), 134.1 (CH-7), 152.0 (C-8), and 201.6 (C-9) in the ^13^C NMR spectrum. Signals of carbons at δ_C_ 133.8 (CH-22) and 133.5 (CH-23) suggested the existence of a disubstituted olefin, and this was confirmed by two olefin proton signals at δ_H_ 5.26 (1H, dd, *J* = 14.8, 6.8 Hz, H-23) and 5.23 (1H, dd, *J* = 14.8, 6.8 Hz, H-22) (Table 1). The presence of Me-21, Me-28, Me-26, and Me-27 groups resulted in four doublets located at δ_H_ 1.05 (3H, *J* = 6.8 Hz), 0.91 (3H, *J* = 6.8 Hz), 0.84 (3H, *J* = 7.2 Hz), and 0.82 (3H, *J* = 7.2 Hz), respectively, and the existence of H_3_-18 and H_3_-19 resulted in two sharp singlets at δ_H_ 0.73 and δ_H_ 1.21, respectively. In the ^1^H NMR spectrum, one acetyl methyl signal (δ_H_ 2.02, 3H, s) was observed. Based on the aforementioned findings, metabolite **1** was determined to be a tricyclic compound.

From the ^1^H NMR spectrum and ^1^H–^1^H correlation spectroscopy (COSY) of **1** (Table 1), the following correlations were revealed: H_2_-1/H_2_-2/H-3/H_2_-4, H_2_-11/H_2_-12, H-14/H_2_-15/H_2_-16/H-17/H-20/H-22/H-23/H-24/H-25/H_3_-26, H-20/H_3_-21, H-24/H_3_-28, and H-25/H_3_-27. Together with data of key heteronuclear multiple bond coherence (HMBC) correlations between H_2_-4, H-7, H_3_-19/C-5; H_2_-4/C-6; H-7, H-14/C-8; H-7, H-14, H_3_-19/C-9; H_2_-1, H_2_-4, H_3_-19/C-10; and H_2_-11, H_2_-12, H-14, and H_3_-18/C-13, all the information allowed determination of the main carbon skeleton of **1** (Table 1).

**Figure 2:**
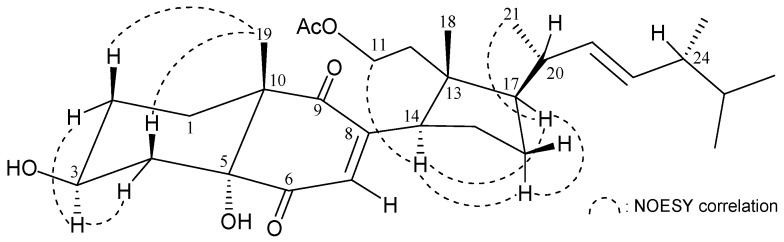
Selected NOESY correlations observed for **1**.

The correlations identified using nuclear Overhauser effect spectroscopy (NOESY) in addition to comparison of NMR data with those of known 9,11-secosterol **8**, isolated from Korean marine sponge *Ircinia* sp. [9], and pinnisterol A (**9**) [4], enabled clarification of the configuration of **1** (Figure 1). The stereochemistries of stereogenic centers C-3, C-5, C-10, C-13, C-14, and C-17 in **1** were the same as those of **8**. In addition, the main NOE correlations for **1** were interactions between H-3/H-2*α* (δ_H_ 2.00), H-2*β* (δ_H_ 1.49)/H_3_-19, H-3/H-4*α* (δ_H_ 2.17), H-4*β* (δ_H_ 1.79)/H_3_-19; thus, H-3 and the 5-hydroxy group in **1** should be positioned on the α-face (Figure 2).

**Figure 3:**
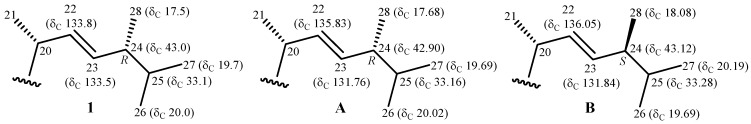
Schematic diagrams of ^13^C NMR chemical shift data of the side-chain of pinnisterol D (**1**), (22*E*,24*R*)-24-methylcholesta-5,22-dien-3*β*-ol (**A**), and (22*E*,24*S*)-24-methylcholesta-5,22-dien-3*β*-ol (**B**) [10].

There was a greater coupling constant between H-22 and H-23 (*J* = 14.8 Hz), which supported a *trans* relationship between H-22 and H-23. This implied that the configuration of C-24 should be *R* according to the ^13^C NMR chemical shift of C-28 (δ_C_ 17.5). A previous study showed that, for a known sterol, (22*E*,24*R*)-24-methylcholesta-5,22-dien-3*β*-ol, with an identical chain, and the 24*S* epimer, (22*E*,24*S*)-24-methylcholesta-5,22-dien-3*β*-ol, the ^13^C NMR value of C-28 resonates at δ_C_ 17.68 ppm in the 24*R* epimer, with a relative 0.4 ppm downfield chemical shift (Figure 3) [10].

**Figure 4:**
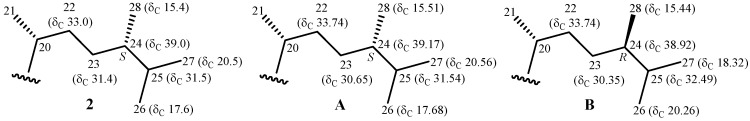
Schematic diagrams of ^13^C NMR chemical shift data of the side-chain of pinnisterol E (**2**), (24*S*)-24-methylcholest-5-en-3*β*-ol (**A**), and (24*R*)-24-methylcholest-5-en-3*β*-ol (**B**) [9].

Pinnisterol E (**2**) was present as a colorless oil. From HRESIMS analysis, the signal at *m/z* 527.33410 (calcd. for C_30_H_48_O_6_ + Na, 527.33486) suggested the molecular formula of **2** to be C_30_H_48_O_6_ (7° of unsaturation), and the IR spectrum demonstrated the existence of hydroxy (ν_max_ 3381 cm^−1^), ester (ν_max_ 1740 cm^−1^), and *α*,*β*-unsaturated ketonic carbonyl (ν_max_ 1686 cm^−1^) groups. The whole series of spectroscopic data demonstrated that secosterols **2** and **1** had an identical core structure, the difference being limited to the absence in **2** of the carbon-carbon double bond between C-22/23. The complete assignments of ^1^H and ^13^C NMR of **2** (Table 2 and Table 3) were compared with the values of **1**, and secosterol **2** was assigned as having structure **2**, with the same configurations of the core rings A–C. In addition, both compounds had identical stereogenic centers at C-3, C-5, C-10, C-13, C-14, and C-17, and their ^1^H and ^13^C NMR chemical shifts and proton coupling constants were in concurrence also. Based on the ^13^C NMR chemical shifts of C-25 (δ_C_ 31.5), C-26 (δ_C_ 17.6), and C-27 (δ_C_ 20.5), the configuration of the stereogenic center at C-24 was assigned as *S*. Previous study also showed that the ^13^C NMR values of C-25, C-26, and C-27 resonates at δ_C_ 31.54, 17.68, and 20.56 ppm in a 24*S* epimer of a known sterol, (24*S*)-24-methylcholest-5-en-3*β*-ol, with an identical side chain, and the ^13^C NMR values of C-25, C-26, and C-27 in a 24*R* epimer, (24*R*)-24-methylcholest-5-en-3*β*-ol, were observed at δ_C_ 32.49, 20.26, and 18.32 ppm, respectively (Figure 4) [10].

Pinnisterol F (**3**) was present as a colorless oil. From HRESIMS analysis, the signal at *m/z* 583.32406 (calcd. for C_32_H_48_O_8_ + Na, 583.32469) suggested the molecular formula of **3** to be C_32_H_48_O_8_ (9° of unsaturation). The NMR signals of **3** (Table 2 and Table 3) were similar to those of **1**, except that the signals related to the C-21 methyl in **1** were substituted by signals for an acetoxymethylene group in **3**. From the HMBC spectrum of **3**, it was revealed that an ester carbonyl carbon at δ_C_ 171.1 correlated with a methyl signal at δ_H_ 2.04 and a pair of oxygenated methylene protons at δ_H_ 4.01 (1H, dd, *J* = 10.5, 7.0 Hz) and 3.96 (1H, dd, *J* = 10.5, 7.0 Hz), which revealed that an acetoxy group was at the position C-21 in the side chain of **3**. Thus, pinnisterol F (**3**) was found to be the 21-acetoxy derivative of **1**.

**Figure 5:**
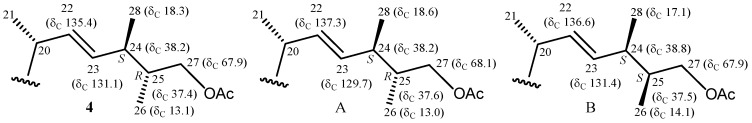
Schematic diagrams of ^13^C NMR chemical shift data of the side-chain of pinnisterol G (**4**), echrebsteroid C (**A**), and echrebsteroid B (**B**) [11].

Pinnisterol G (**4**) had a molecular formula identical to that of **3**, C_32_H_48_O_8_, with a HRESIMS signal located at *m/z* 583.32432 (calcd. for C_32_H_48_O_8_ + Na, 583.32469) with nine degrees of unsaturation, indicating that secosterols **3** and **4** were isomers. Comparison of the NMR data of **4** with those of **3** (Table 2 and Table 3) showed that both compounds possessed the same sterol nucleus and a similar side chain, but differed in terms of the location of one acetoxy group. From an HMBC experiment, it was revealed that one ester carbonyl carbon at δ_C_ 171.3 correlated with one methyl signal at δ_H_ 2.07 and a pair of oxymethylene protons signals at δ_H_ 3.98 (dd, *J* = 10.5, 6.3 Hz) and 3.85 (dd, *J* = 10.5, 6.3 Hz), which indicated that an acetoxy group was located at C-27 in the side chain. The configurations at C-24 and C-25 were therefore designated as *S*- and *R*-forms, respectively, on the basis of the ^13^C NMR chemical shifts of C-24 (δ_C_ 38.2), C-25 (δ_C_ 37.4), C-26 (δ_C_ 13.1), C-27 (δ_C_ 67.9), and C-28 (δ_C_ 18.3). It was reported that the ^13^C NMR values of C-24, C-25, C-26, C-27, and C-28 resonate at δ_C_ 38.2, 37.6, 13.0, 68.1, and 18.6 ppm in a 24*S* and a 25*R* epimer of a known sterol, echrebsteroid C, with the same side chain, and the ^13^C NMR values of C-24, C-25, C-26, C-27, and C-28 in a 24*S* and 25*S* epimer, echrebsteroid B, appeared at *δ*_C_ 38.8, 37.5, 14.1, 67.9, and 17.1 ppm (Figure 5) [11].

Pinnisterol H (**5**) was isolated as a colorless oil. Based on the HRESIMS signal located at *m/z* 585.33988 (calcd. for C_32_H_50_O_8_ + Na, 585.34034), it was concluded that the molecular formula of **5** was C_32_H_50_O_8_ (8° of unsaturation). The IR spectrum of **5** indicated the presence of hydroxy (ν_max_ 3448 cm^−1^), ester (ν_max_ 1736 cm^−1^) and *α*,*β*-unsaturated ketonic carbonyl (ν_max_ 1686 cm^−1^) groups. The whole series of spectroscopic data showed that secosterol **5** and secosterol **1** shared the same core structure, with the exception of the addition of an acetoxy group to substitute the alkene at C-23 in **5**. The complete assignments of the ^13^C and ^1^H NMR of pinnisterol H (**5**) (Table 3 and Table 4) were compared with the values of **1**, and the HMBC correlations fully supported the positions of the functional groups of **5**, indicating that it had a structure of the same configuration as secosterols **1**–**4** in the core rings A–C. The proton coupling constants and NMR chemical shift data also further supported this finding, though the configurations of C-23 and C-24 were not determined at this stage.

Pinnisterol I (**6**) was obtained as a colorless oil. The HRESIMS signal at *m/z* 585.33988 (calcd. for C_32_H_50_O_8_ + Na, 585.34034) suggested the molecular formula of **6** to be C_32_H_50_O_8_ (8° of unsaturation). The NMR signals of **6** (Table 3 and Table 4) were very similar to those of **5**, with the exception that **5** had signals corresponding to 3-hydroxy and 23-acetoxy groups, which were substituted by signals for acetoxy and hydroxy groups, respectively, in **6**. From a NOESY experiment, the correlations of data of **5** and **6** demonstrated that the configurations of the stereogenic centers in the core rings A–C were identical to those of **1**. The configurations of stereogenic centers C-23 and C-24 of **6** were also not determined at this stage.

**Figure 6:**
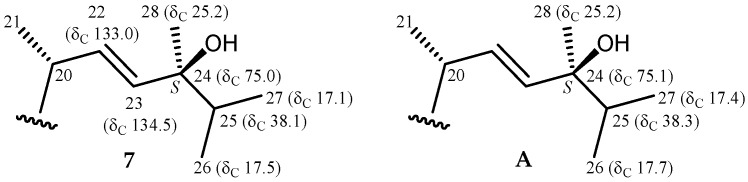
Schematic diagrams of ^13^C NMR chemical shift data of the side-chain of pinnisterol J (**7**) and 24(*S*)-hydroxyvitamin D_2_ (**A**) [12].

Pinnisterol J (**7**) was obtained as a colorless oil and had the molecular formula C_32_H_48_O_8_, as determined by the HRESIMS signal at *m/z* 583.32433 (calcd. for C_32_H_48_O_8_ + Na, 583.32469) (9° of unsaturation). According to the NMR spectroscopic data (Table 3 and Table 4), compound **7** showed the same nuclear structure as that of compound **6**. In the ^13^C NMR data of **7**, one additional disubstituted olefin was identified from signals of carbons at δ_C_ 132.9 (CH-22) and 134.5 (CH-23). The presence of a 24-hydroxy group was evidenced by HMBC correlations between H-22, H-23, H-25, H_3_-26, H_3_-27, and H_3_-28/C-24 (δ_C_ 75.0), a methyl-containing oxygenated tertiary carbon. There was a greater coupling constant between H-22 and H-23 (*J* = 15.6 Hz), suggesting that a *trans* relationship existed between H-22 and H-23. The configuration of the C-24 stereogenic center was assigned as *S* on the basis of the ^1^^3^C NMR chemical shifts of C-24 (δ_C_ 75.0), C-25 (δ_C_ 38.1), C-26 (δ_C_ 17.5), C-27 (δ_C_ 17.1), and C-28 (δ_C_ 25.2). It was reported that the ^1^^3^C NMR values of C-24, C-25, C-26, C-27, and C-28 resonates at δ_C_ 75.1, 38.3, 17.7, 17.4, and 25.2 ppm in a 24*S* epimer of a known synthetic product, 24(*S*)-hydroxyvitamin D_2_, with the same side chain (Figure 6) [12].

**Figure 7:**
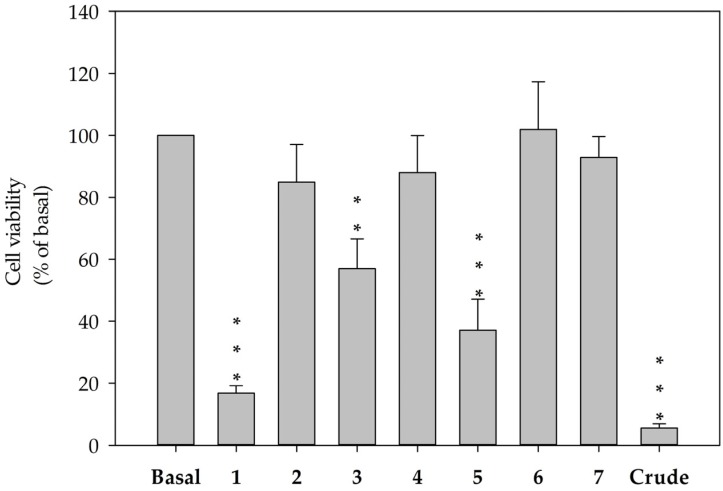
Decreased cell viability in HSC-T6 cells treated with secosterols **1**–**7** for 24 h at a concentration of 10 μM. Cells were treated with secosterols, DMSO (control) or coral crude extract at 6 μg/mL. Cytotoxicity was monitored spectrophotometrically at OD 450 nm. Quantitative data are expressed as the mean ± S.E.M. (*n* = 3–4). ** *p* < 0.01, *** *p* < 0.001 compared to basal.

The hepatic stellate cell, a major cell type involved in liver fibrosis, is also responsible for liver damage by increasing proliferation and protein secretion associated with the formation of scar tissue. In cytotoxicity testing, secosterols **1**–**7** were examined in terms of their cytotoxic effects on HSC-T6, an immortalized rat hepatic stellate cell lines. At a concentration of 10 μM, secosterols **1**, **3**, and **5** significantly decreased the viability of HSC-T6 cells to 16.8 (IC_50_ = 3.93 μM), 56.9 and 37.1%, respectively (Figure 7). These results implied that the functional groups in the side chain of secosterols **1**–**7** play important roles in determining the activity of the compounds.

In anti-inflammatory testing, secosterols **2**, **5**, and **7** displayed inhibitory effects on the release of elastase (IC_50_ = 2.33, 2.59 and 3.89 μM, respectively), and secosterols **3**, **5**, and **7** showed inhibitory effects on human neutrophils in terms of the generation of superoxide anions (IC_50_ = 5.52, 3.26, and 3.71 μM, respectively) (Table 5).

## 3. Experimental Section

### 3.1. General Experimental Procedures

Optical rotations were measured with a digital polarimeter (P-1010; Japan Spectroscopic Corporation, Tokyo, Japan); and infrared spectra were recorded on a spectrometer (FT/IR-4100; Japan Spectroscopic Corporation); peaks are reported in cm^−1^. NMR spectra were obtained on a 400 MHz NMR spectrometer (Mercury Plus; Varian, Palo Alto, CA, USA) and a 700 MHz NMR spectrometer (AVIIIHD700X; Bruker, Bremen, Germany), using the residual CHCl_3_ signal (δ_H_ 7.26 ppm) and CDCl_3_ (δ_C_ 77.1 ppm) as internal standards for ^1^H NMR and ^13^C NMR, respectively. Coupling constant values (*J*) are given in Hz. ESIMS and HRESIMS were performed using mass spectrometry (Tesla solariX FTMS system, Bruker). TLC was carried out on Kieselgel 60 F_254_ precoated plates (0.25 mm, Merck, Darmstadt, Germany), and spots were visualized by the standard method. Column chromatography was performed on silica gel at a size of 230–400 mesh (Merck). HPLC experiments were performed using the following systems: normal-phase HPLC (NP-HPLC) injection port, 7725 (Rheodyne, Rohnert Park, CA, USA); pump, L-7110 (Hitachi, Tokyo, Japan); and semi-preparative normal-phase column (Supelco Ascentis Si Cat #:581515-U, 25 cm × 21.2 mm, 5 μm, Sigma-Aldrich, St. Louis, MO, USA). Reverse-phase HPLC (RP-HPLC) injection port (7725; Rheodyne); pump, L-2130 (Hitachi); photodiode array detector (L-2455; Hitachi); and reverse-phase column (25 cm × 21.2 mm, Luna 5 μm C18(2) 100 Å, AXIA Packed; Phenomenex, Torrance, CA, USA).

### 3.2. Animal Material

Specimens of gorgonian coral *Pinnigorgia* sp. were collected in August 2012 by hand while scuba diving off the coast of Green Island located near the southeast of Taiwan. The samples were then stored in a freezer until extraction. A voucher specimen was deposited in the National Museum of Marine Biology & Aquarium, Taiwan (specimen No.: NMMBA-TW-GC-2012-130). Identification of the species of this organism was done by comparison as described in the previous publication [13].

### 3.3. Extraction and Separation

Extraction of compounds was performed at room temperature unless otherwise specified. *Pinnigorgia* sp. (wet weight 1.98 kg; dry weight 0.86 kg) was sliced, and the sliced bodies were then extracted with ethyl acetate (EtOAc). The EtOAc extract (84.9 g) was partitioned with *n*-hexane and methanol (MeOH). The MeOH layer (12.6 g) was separated on a Sephadex LH-20 column and elution was performed using a solvent mixture dichloromethane (DCM):MeOH (1:1); the separation yielded 7 subfractions A–G. Fraction F was separated by silica gel column chromatography and then eluted with *n*-hexane/acetone (stepwise, 50/50 %v/v to 100% acetone) to afford eight subfractions F1–F8. Fraction F2 was purified by silica gel column chromatography and then eluted with *n*-hexane/acetone (stepwise, 90/10 %v/v to 100% acetone) to yield 13 subfractions F2A–F2M. Fraction F2F was purified by NP-HPLC using a mixture of *n*-hexane/EtOAc (5:2) to afford 14 subfractions F2F1–F2F14. Fractions F2F5 and F2F6 were repurified by RP-HPLC using a mixture of MeOH/H_2_O (90/10 %v/v at 5.0 mL/min flow rate) to yield **6** (1.8 mg) and **7** (4.8 mg), respectively. Fraction F2H was purified by NP-HPLC using a mixture of *n*-hexane/EtOAc (50/50 %v/v) to yield 14 subfractions F2H1–F2H14. Fractions F2H9 and F2H10 were repurified by RP-HPLC using a mixture of MeOH/H_2_O (90/10 %v/v at 5.0 mL/min flow rate) to yield **1** (3.8 mg) and **2** (1.5 mg), respectively. Fraction F2I was repurified by NP-HPLC using a mixture of *n*-hexane/EtOAc (50/50 %v/v at 3.0 mL/min flow rate) to afford 15 subfractions F2I1–F2I15, including compound **5** (F2I9, 6.4 mg). Fraction F2I10 was purified by RP-HPLC using a mixture of MeOH/H_2_O (85/15 %v/v at 5.0 mL/min, flow rate) to afford **3** (1.0 mg) and **4** (1.0 mg), respectively.

Pinnisterol D (**1**): colorless oil; [α]${}_{D}^{25}$ +44 (*c* 1.3, CHCl_3_); IR (neat) ν_max_ 3446, 1741, 1685 cm^−^^1^; ^1^H (400 MHz, CDCl_3_) and ^13^C (100 MHz, CDCl_3_) NMR data (see Table 1); ESIMS *m*/*z* 525 [M + Na]^+^; HRESIMS *m*/*z* 525.31853 (calcd. for C_30_H_4__6_O_6_ + Na, 525.31921).

Pinnisterol E (**2**): colorless oil; [α]${}_{D}^{25}$ −35 (*c* 0.5, CHCl_3_); IR (neat) ν_max_ 3381, 1740, 1686 cm^−^^1^; ^1^H (400 MHz, CDCl_3_) and ^13^C (100 MHz, CDCl_3_) NMR data (see Table 2 and Table 3); ESIMS *m*/*z* 527 [M + Na]^+^; HRESIMS *m*/*z* 527.33410 (calcd. for C_3__0_H_48_O_6_ + Na, 527.33486).

Pinnisterol F (**3**): colorless oil; [α]${}_{D}^{27}$ −152 (*c* 0.3, CHCl_3_); IR (neat) ν_max_ 3446, 1736, 1686 cm^−^^1^; ^1^H (700 MHz, CDCl_3_) and ^13^C (175 MHz, CDCl_3_) NMR data (see Table 2 and Table 3); ESIMS *m*/*z* 583 [M + Na]^+^; HRESIMS *m*/*z* 583.32406 (calcd. for C_3__2_H_48_O_8_ + Na, 583.32469).

Pinnisterol G (**4**): colorless oil; [α]${}_{D}^{27}$ −176 (*c* 0.3, CHCl_3_); IR (neat) ν_max_ 3447, 1739, 1686 cm^−^^1^; ^1^H (700 MHz, CDCl_3_) and ^13^C (175 MHz, CDCl_3_) NMR data (see Table 2 and Table 3); ESIMS *m*/*z* 583 [M + Na]^+^; HRESIMS *m*/*z* 583.32432 (calcd. for C_3__2_H_48_O_8_ + Na, 583.32469).

Pinnisterol H (**5**): colorless oil; [α]${}_{D}^{25}$ −20 (*c* 0.3, CHCl_3_); IR (neat) ν_max_ 3448, 1736, 1686 cm^−^^1^; ^1^H (400 MHz, CDCl_3_) and ^13^C (100 MHz, CDCl_3_) NMR data (see Table 3 and Table 4); ESIMS *m*/*z* 585 [M + Na]^+^; HRESIMS *m*/*z* 585.33988 (calcd. for C_32_H_5__0_O_8_ + Na, 585.34034).

Pinnisterol I (**6**): colorless oil; [α]${}_{D}^{25}$ −129 (*c* 0.5, CHCl_3_); IR (neat) ν_max_ 3446, 1736, 1685 cm^−^^1^; ^1^H (400 MHz, CDCl_3_) and ^13^C (100 MHz, CDCl_3_) NMR data (see Table 3 and Table 4); ESIMS *m*/*z* 585 [M + Na]^+^; HRESIMS *m*/*z* 585.33988 (calcd. for C_3__2_H_5__0_O_8_ + Na, 585.34034).

Pinnisterol J (**7**): colorless oil; [α]${}_{D}^{25}$ −5 (*c* 0.2, CHCl_3_); IR (neat) ν_max_ 3455, 1736, 1687 cm^−^^1^; ^1^H (400 MHz, CDCl_3_) and ^13^C (100 MHz, CDCl_3_) NMR data (see Table 3 and Table 4); ESIMS *m*/*z* 583 [M + Na]^+^; HRESIMS *m*/*z* 583.32433 (calcd. for C_32_H_48_O_8_ + Na, 583.32469).

### 3.4. Anti-Hepatofibric Assay

The anti-hepatofibric effects of secosterols **1**–**7**, at a concentration of 10 μM, were analyzed using a colorimetric assay (WST-1-based method). The steps of the assay were performed according to a previously-published method [14].

### 3.5. Generation of Superoxide Anions and Release of Elastase by Human Neutrophils

Human neutrophils were prepared by Ficoll centrifugation followed by dextran sedimentation. Measurements of superoxide anion generation and elastase release from neutrophils were performed based on published procedures [15,16]. Briefly, MeO-Suc-Ala-Ala-Pro-Valp-nitroanilide was used as the elastase substrate for the elastase release assay, and the method using superoxide dismutase-inhibitable reduction of ferricytochrome *c* was employed to measure superoxide anion production.

## 4. Conclusions

Our ongoing investigations of coral metabolites showed that gorgonian corals belonging to the genus *Pinnigorgia* are rich in 9,11-secosterols. In cytotoxicity tests, secosterol **1** showed significant cytotoxicity against HSC-T6 cells. In anti-inflammatory activity tests on human neutrophils, secosterols **2**, **5**, and **7** displayed inhibitory effects on elastase release, and **3**, **5**, and **7** showed inhibitory effects on the generation of superoxide anions. Our findings suggested that these new 9,11-secosterols could be developed as promising bioactive agents, and further biomedical study is necessary in order to identify their potential applications in the treatment of disease.

## Figures and Tables

**Table 1:** Results of ^1^H (400 MHz, CDCl_3_) and ^13^C (100 MHz, CDCl_3_) NMR experiments and ^1^H–^1^H COSY and HMBC correlations for secosterol **1**.

Position	δ_H_ (*J* in Hz)	δ_C_, Multiple	^1^H–^1^H COSY	HMBC
1	2.21 m; 1.78 m	25.9, CH_2_	H_2_-2	C-2, -10
2	2.00 m; 1.49 m	29.9, CH_2_	H_2_-1, H-3	n. o. ^a^
3	4.03 m	66.7, CH	H_2_-2, H_2_-4	n. o.
4	2.17 m; 1.79 m	35.7, CH_2_	H-3	C-2, -3, -5, -6, -10
5		80.6, C		
6		197.5, C		
7	6.45 s	134.1, CH		C-5, -8, -9, -14
8		152.0, C		
9		201.6, C		
10		52.0, C		
11	4.18 m	61.0, CH_2_	H_2_-12	C-12, -13, acetate carbonyl
12	1.68 m; 1.26 ddd (14.8, 9.2, 5.6)	37.1, CH_2_	H_2_-11	C-11, -13, -14, -17, -18
13		47.4, C		
14	3.37 dd (10.8, 8.0)	43.9, CH	H_2_-15	C-7, -8, -9, -13, -15, -18
15	1.75 m; 1.66 m	27.1, CH_2_	H-14, H_2_-16	C-16
16	1.78 m; 1.57 m	25.6, CH_2_	H_2_-15, H-17	C-15
17	1.81 m	51.0, CH	H_2_-16, H-20	C-15, -16, -18, -21
18	0.73 s	18.0, CH_3_		C-12, -13, -14, -17
19	1.21 s	20.5, CH_3_		C-1, -5, -9, -10
20	2.24 m	38.3, CH	H-17, H_3_-21, H-22	C-16, -17, -22, -23
21	1.05 d (6.8)	21.8, CH_3_	H-20	C-17, -20, -22
22	5.23 dd (14.8, 6.8)	133.8, CH	H-20, H-23	C-20, -24
23	5.26 dd (14.8, 6.8)	133.5, CH	H-22, H-24	C-20, -24
24	1.86 m	43.0, CH	H-23, H-25, H_3_-28	C-22, -23, -25, -26, -27, -28
25	1.46 m	33.1, CH	H-24, H_3_-26, H_3_-27	C-23, -24, -26, -27, -28
26	0.84 d (7.2)	20.0, CH_3_	H-25	C-24, -25, -27
27	0.82 d (7.2)	19.7, CH_3_	H-25	C-24, -25, -26
28	0.91 d (6.8)	17.5, CH_3_	H-24	C-23, -24, -25
11-OAc		171.1, C		
	2.02 s	21.1, CH_3_		Acetate carbonyl

^a^ n. o. = not observed.

**Table 2:** ^1^H NMR data for secosterols **2**–**4**.

δ_H_	2 ^a^	3 ^b^	4 ^b^
1	2.21 m; 1.76 m	2.21 m; 1.74 m	2.20 m; 1.72 m
2	2.00 m; 1.47 m	2.00 m; 1.47 m	2.00 m; 1.49 m
3	4.04 m	4.04 m	4.04 m
4	2.15 ddd (14.4, 5.2, 2.0) ^c^; 1.79 m	2.14 m; 1.77 m	2.13 m; 1.79 m
7	6.47 s	6.46 s	6.46 s
11	4.16 m	4.17 t (6.3)	4.18 m
12	1.73 m; 1.23 m	1.59 m; 1.30 m	1.69 m; 1.25 m
14	3.39 dd (10.8, 8.4)	3.37 t (9.8)	3.38 dd (11.2, 8.4)
15	1.76 m; 1.68 m	1.77 m; 1.69 m	1.76 m; 1.65 m
16	1.89 m; 1.53 m	1.75 m; 1.49 m	1.76 m; 1.55 m
17	1.76 m	1.97 m	1.77 m
18	0.74 s	0.73 s	0.73 s
19	1.22 s	1.21 s	1.22 s
20	1.42 m	1.46 m	2.23 m
21	0.99 d (6.8)	4.01 dd (10.5, 7.0); 3.96 dd (10.5, 7.0)	1.06 d (7.0)
22	1.44 m; 0.95 m	5.36 dd (15.4, 8.4)	5.24 dd (15.4, 8.4)
23	1.40 m; 0.96 m	5.22 dd (15.4, 8.4)	5.30 dd (15.4, 8.4)
24	1.20 m	1.90 m	2.19 m
25	1.61 m	1.45 m	1.71 m
26	0.79 d (6.8)	0.84 d (7.0)	0.85 d (7.0)
27	0.86 d (6.8)	0.82 d (7.0)	3.98 dd (10.5, 6.3); 3.85 dd (10.5, 6.3)
28	0.78 d (6.8)	0.93 d (7.0)	0.98 d (7.0)
11-OAc	2.02 s	2.02 s	2.03 s
21-OAc		2.04 s	
27-OAc			2.07 s

^a^ Spectra recorded at 400 MHz in CDCl_3_; ^b^ Spectra recorded at 700 MHz in CDCl_3_; ^c^ *J* values (in Hz) in parentheses.

**Table 3:** ^13^C NMR data for secosterols **2**–**7**.

δ_C_	2 ^a^	3 ^b^	4 ^b^	5 ^a^	6 ^a^	7 ^a^
1	25.9, CH_2_	25.8, CH_2_	25.9, CH_2_	25.9, CH_2_	25.7, CH_2_	25.7, CH_2_
2	29.9, CH_2_	29.9, CH_2_	29.9, CH_2_	29.9, CH_2_	25.9, CH_2_	25.9, CH_2_
3	66.7, CH	66.7, CH	66.7, CH	66.7, CH	69.7, CH	69.8, CH
4	35.8, CH_2_	35.7, CH_2_	35.7, CH_2_	35.6, CH_2_	32.0, CH_2_	31.9, CH_2_
5	80.6, C	80.6, C	80.6, C	80.5, C	80.1, C	80.0, C
6	197.4, C	197.4, C	197.5, C	197.5, C	197.3, C	197.5, C
7	134.1, CH	134.1, CH	134.1, CH	134.2, CH	134.2, CH	134.2, CH
8	152.1, C	151.6, C	151.8, C	151.7, C	151.8, C	151.6, C
9	201.5, C	201.6, C	201.6, C	201.6, C	201.2, C	201.3, C
10	52.0, C	52.0, C	52.0, C	52.0, C	51.9, C	51.9, C
11	61.0, CH_2_	60.8, CH_2_	61.0, CH_2_	60.9, CH_2_	61.0, CH_2_	61.0, CH_2_
12	37.2, CH_2_	36.7, CH_2_	37.0, CH_2_	37.0, CH_2_	37.2, CH_2_	37.2, CH_2_
13	47.5, C	47.5, C	47.3, C	47.4, C	47.4, C	47.4, C
14	43.7, CH	43.5, CH	43.8, CH	43.7, CH	43.7, CH	43.8, CH
15	27.2, CH_2_	27.1, CH_2_	26.9, CH_2_	27.0, CH_2_	27.1, CH_2_	27.1, CH_2_
16	26.2, CH_2_	25.8, CH_2_	25.7, CH_2_	26.2, CH_2_	26.4, CH_2_	25.8, CH_2_
17	50.7, CH	46.3, CH	50.7, CH	51.4, CH	51.6, CH	50.9, CH
18	17.6, CH_3_	18.5, CH_3_	18.0, CH_3_	17.6, CH_3_	17.8, CH_3_	18.0, CH_3_
19	20.5, CH_3_	20.4, CH_3_	20.5, CH_3_	20.4, CH_3_	20.3, CH_3_	20.2, CH_3_
20	34.9, CH	42.2, CH	38.4, CH	33.2, CH	33.9, CH	38.2, CH
21	19.2, CH_3_	67.2, CH_2_	21.7, CH_3_	20.4, CH_3_	21.1, CH_3_	21.6, CH_3_
22	33.0, CH_2_	127.5, CH	135.4, CH	35.4, CH_2_	39.4, CH_2_	132.9, CH
23	21.4, CH_2_	138.1, CH	131.1, CH	76.7, CH	74.6, CH	134.5, CH
24	39.0, CH	43.4, CH	38.2, CH	42.7, CH	45.5, CH	75.0, C
25	31.5, CH	32.9, CH	37.4, CH	28.5, CH	27.7, CH	38.1, CH
26	17.6, CH_3_	20.0, CH_3_	13.1, CH_3_	21.6, CH_3_	21.7, CH_3_	17.5, CH_3_
27	20.5, CH_3_	19.7, CH_3_	67.9, CH_2_	18.6, CH_3_	17.6, CH_3_	17.1, CH_3_
28	15.4, CH_3_	17.5, CH_3_	18.3, CH_3_	11.0, CH_3_	10.5, CH_3_	25.2, CH_3_
3-OAc					170.8, C	170.9, C
					21.3, CH_3_	21.1, CH_3_
11-OAc	171.1, C	171.1, C	171.1, C	171.1, C	171.0, C	170.9, C
	21.1, CH_3_	21.1, CH_3_	21.1, CH_3_	21.1, CH_3_	21.1, CH_3_	21.1, CH_3_
21-OAc		171.1, C				
		21.0, CH_3_				
23-OAc				170.7, C		
				21.4, CH_3_		
27-OAc			171.3, C			
			21.1, CH_3_			

^a^ Spectra recorded at 100 MHz in CDCl_3_; ^b^ Spectra recorded at 175 MHz in CDCl_3_.

**Table 4:** ^1^H NMR (400 MHz, CDCl_3_) data for secosterols **5**–**7**.

δ_H_	5	6	7
1	2.22 m; 1.76 m	2.27 m; 1.78 m	2.28 m; 1.76 m
2	1.97 m; 1.48 m	1.98 m; 1.60 m	1.96 m; 1.61 m
3	4.02 m	5.05 m	5.05 m
4	2.12 m; 1.78 m	2.24 m; 1.81 m	2.24 m; 1.79 m
7	6.45 s	6.46 s	6.45 s
11	4.14 m	4.18 m	4.17 m
12	1.63 m; 1.23 m	1.70 m; 1.27 m	1.67 m; 1.26 m
14	3.36 dd (11.2, 8.4) ^a^	3.39 dd (10.8, 8.0)	3.39 dd (10.4, 8.0)
15	1.76 m; 1.65 m	1.80 m; 1.69 m	1.76 m; 1.69 m
16	1.91 m; 1.52 m	1.93 m; 1.53 m	1.99 m; 1.53 m
17	1.69 m	1.81 m	1.81 m
18	0.73 s	0.74 s	0.73 s
19	1.20 s	1.22 s	1.21 s
20	1.54 m	1.63 m	2.28 m
21	1.02 d (6.8)	1.13 d (6.8)	1.08 d (6.8)
22	1.67 m; 1.24 m	1.72 m; 1.07 m	5.53 dd (15.6, 8.4)
23	5.01 m	3.59 m	5.48 (15.6)
24	1.47 m	1.32 m	
25	1.57 m	1.84 m	1.67 m
26	0.93 d (6.8)	0.94 d (7.2)	0.89 d (6.8)
27	0.83 d (6.8)	0.84 d (7.2)	0.88 d (6.8)
28	0.81 d (6.8)	0.79 d (7.2)	1.21 s
3-OAc		2.04 s	2.04 s
11-OAc	2.02 s	2.02 s	2.02 s
23-OAc	2.03 s		

^a^ *J* values (in Hz) in parentheses.

**Table 5:** Inhibitory effects of secosterols **2**–**7** on elastase release and superoxide anion generation by human neutrophils in response to fMet-Leu-Phe/Cytochalastin B.

Compound	Elastase Release	Superoxide Anions
	IC_50_ (μM)	IC_50_ (μM)
**2**	2.33 ± 0.27	>10
**3**	>10	5.52 ± 1.06
**4**	>10	>10
**5**	2.59 ± 0.29	3.26 ± 0.33
**6**	>10	>10
**7**	3.89 ± 1.16	3.71 ± 0.51

## Supplementary Materials

HRESIMS, ^1^H and ^13^C spectra of new compounds **1**–**7** and 2D NMR data (HSQC, ^1^H−^1^H COSY, HMBC and NOESY spectra) of new compound **1** are available online at www.mdpi.com/1660-3397/15/1/11/s1.
