# Supplementary material for: Pinnisterols D–J, New 11-Acetoxy-9,11-secosterols with a 1,4-Quinone Moiety from Formosan Gorgonian Coral Pinnigorgia sp. (Gorgoniidae)
Source: Mar Drugs. 2017 Jan 6;15(1):11. doi: 10.3390/md15010011 (PMC5295231; doi:10.3390/md15010011)
Supplement: Supplementary file 1 [file marinedrugs-15-00011-s001.docx]

Supplementary Materials: **Pinnisterols D–J, New**
**11-**Acetoxy-9,11-secosterols with a 1,4-Quinone Moiety from Formosan Gorgonian Coral
*Pinnigorgia* sp. (Gorgoniidae)

Yu-Chia Chang, Tsong-Long Hwang, Liang-Mou Kuo and Ping-Jyun Sung

| **No.** | **Content** | **Page** |
| --- | --- | --- |
| Figure S1. | HRESIMS spectrum of compound **1**. | S2 |
| Figure S2. | ^1^H NMR spectrum (400 MHz) of compound **1** in CDCl_3_. | S2 |
| Figure S3. | ^13^C NMR spectrum (100 MHz) of compound **1** in CDCl_3_. | S3 |
| Figure S4. | gHSQC spectrum (400 MHz) of compound **1** in CDCl_3_. | S3 |
| Figure S5. | ^1^H–^1^H COSY spectrum (400 MHz) of compound **1** in CDCl_3_. | S4 |
| Figure S6. | gHMBC spectrum (400 MHz) of compound **1** in CDCl_3_. | S4 |
| Figure S7. | NOESY spectrum (400 MHz) of compound **1** in CDCl_3_. | S5 |
| Figure S8. | HRESIMS spectrum of compound **2**. | S5 |
| Figure S9. | ^1^H NMR spectrum (400 MHz) of compound **2** in CDCl_3_. | S6 |
| Figure S10. | ^13^C NMR spectrum (100 MHz) of compound **2** in CDCl_3_. | S6 |
| Figure S11. | HRESIMS spectrum of compound **3**. | S7 |
| Figure S12. | ^1^H NMR spectrum (700 MHz) of compound **3** in CDCl_3_. | S7 |
| Figure S13. | ^13^C NMR spectrum (175 MHz) of compound **3** in CDCl_3_. | S8 |
| Figure S14. | HRESIMS spectrum of compound **4**. | S8 |
| Figure S15. | ^1^H NMR spectrum (700 MHz) of compound **4** in CDCl_3_. | S9 |
| Figure S16. | ^13^C NMR spectrum (175 MHz) of compound **4** in CDCl_3_. | S9 |
| Figure S17. | HRESIMS spectrum of compound **5**. | S10 |
| Figure S18. | ^1^H NMR spectrum (400 MHz) of compound **5** in CDCl_3_. | S10 |
| Figure S19. | ^13^C NMR spectrum (100 MHz) of compound **5** in CDCl_3_. | S11 |
| Figure S20. | HRESIMS spectrum of compound **6**. | S11 |
| Figure S21. | ^1^H NMR spectrum (400 MHz) of compound **6** in CDCl_3_. | S12 |
| Figure S22. | ^13^C NMR spectrum (100 MHz) of compound **6** in CDCl_3_. | S12 |
| Figure S23. | HRESIMS spectrum of compound **7**. | S13 |
| Figure S24. | ^1^H NMR spectrum (400 MHz) of compound **7** in CDCl_3_. | S13 |
| Figure S25. | ^13^C NMR spectrum (100 MHz) of compound **7** in CDCl_3_. | S14 |


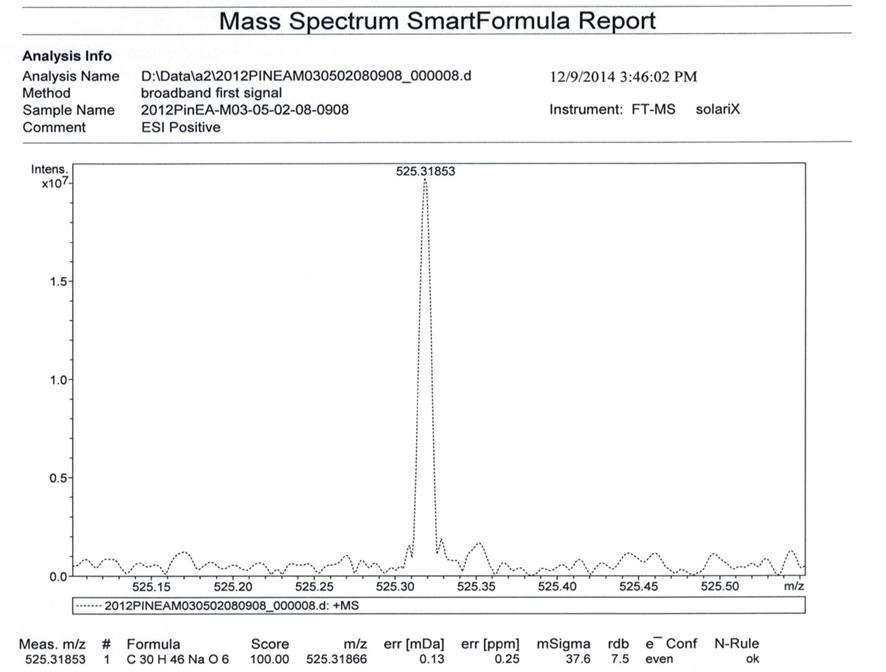


**Figure S1.** HRESIMS spectrum of compound **1.**


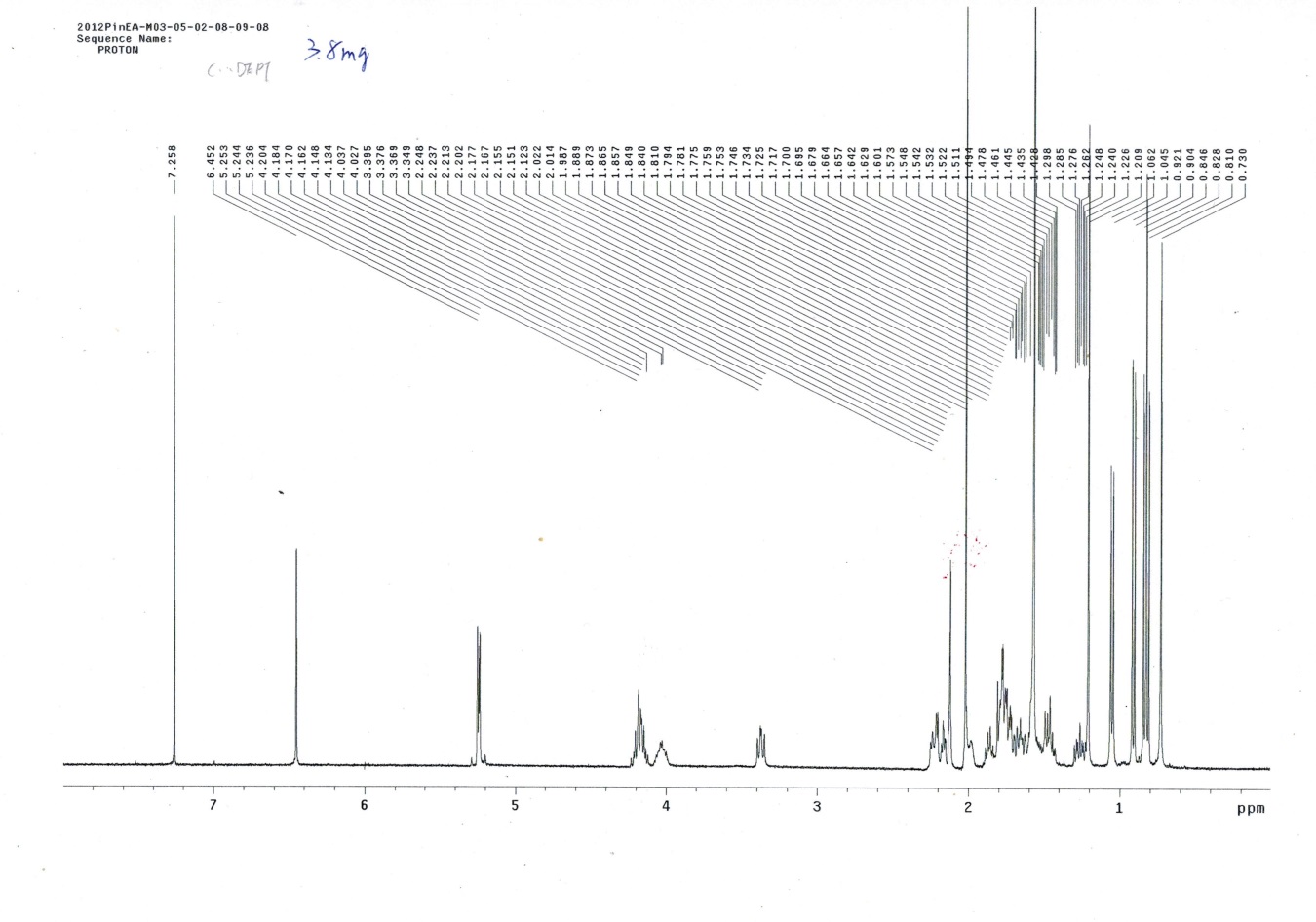


**Figure S2.** ^1^H NMR spectrum (400 MHz) of compound **1** in CDCl_3_.


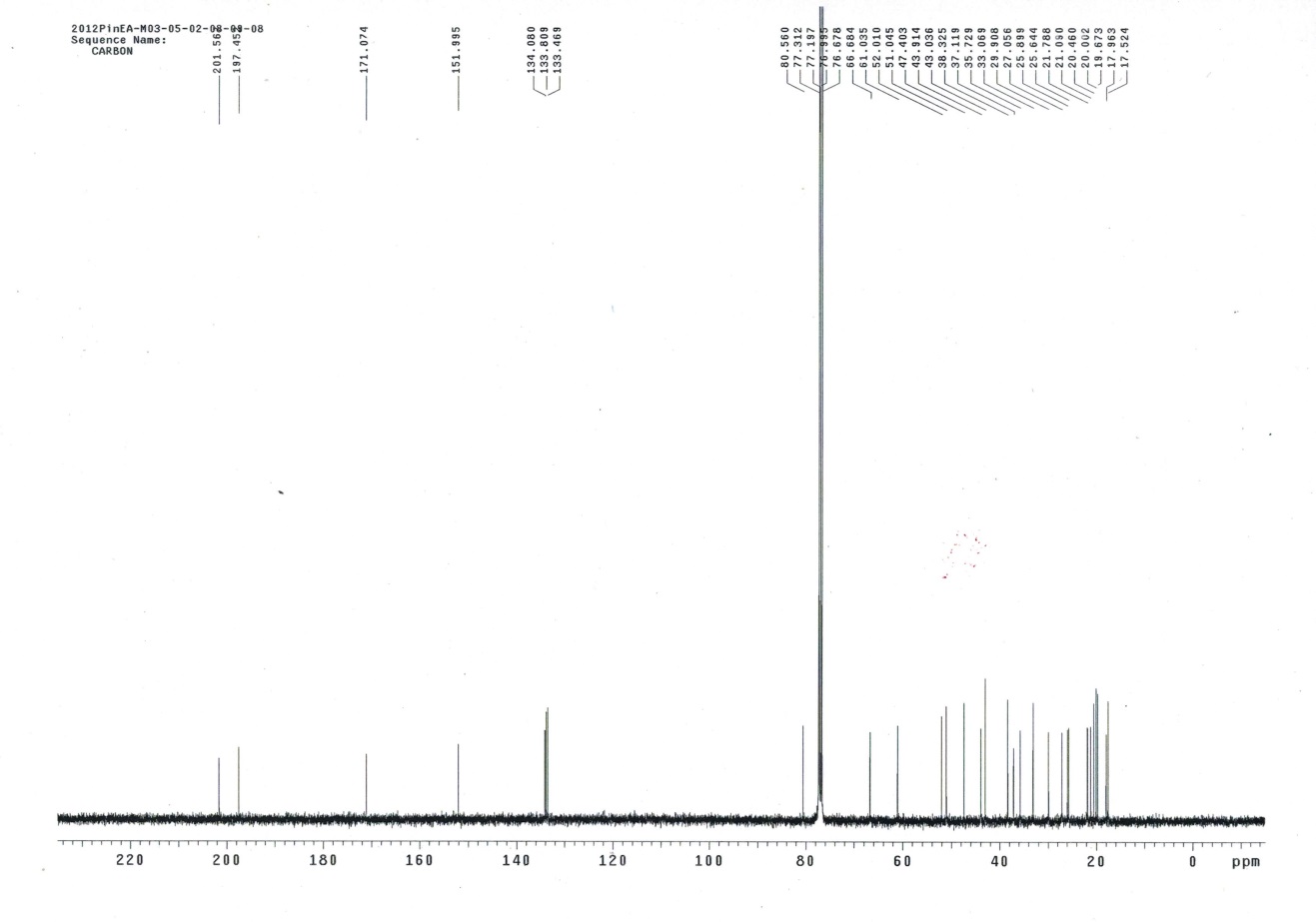


**Figure S3.** ^13^C NMR spectrum (100 MHz) of compound **1** in CDCl_3_.


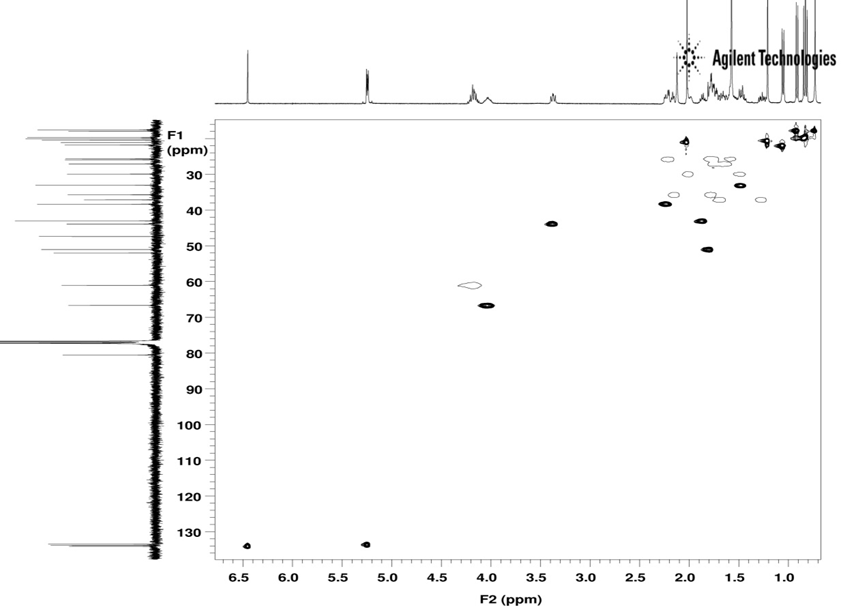


**Figure S4.** gHSQC spectrum (400 MHz) of compound **1** in CDCl_3_.


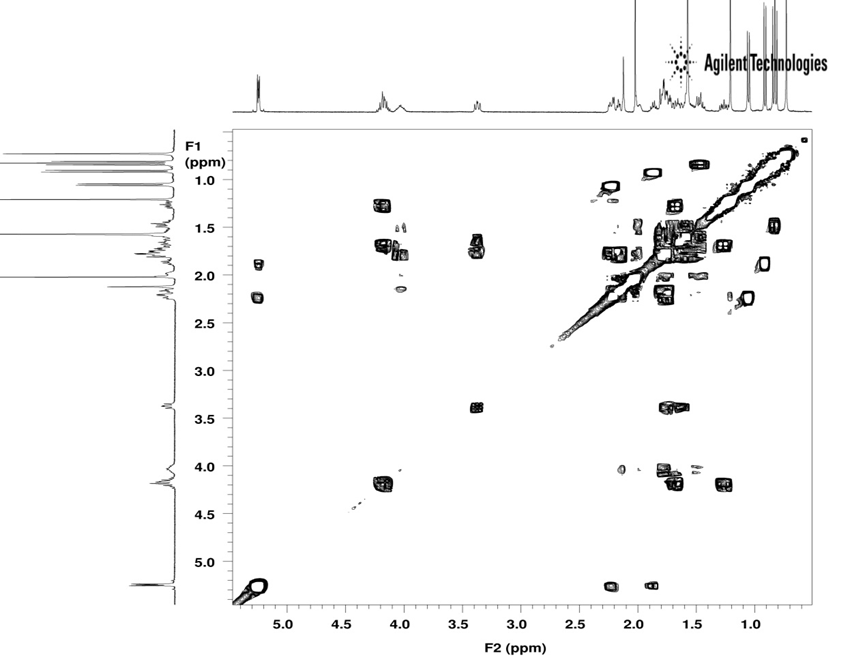


**Figure S5.** ^1^H−^1^H COSY spectrum (400 MHz) of compound **1** in CDCl_3_.


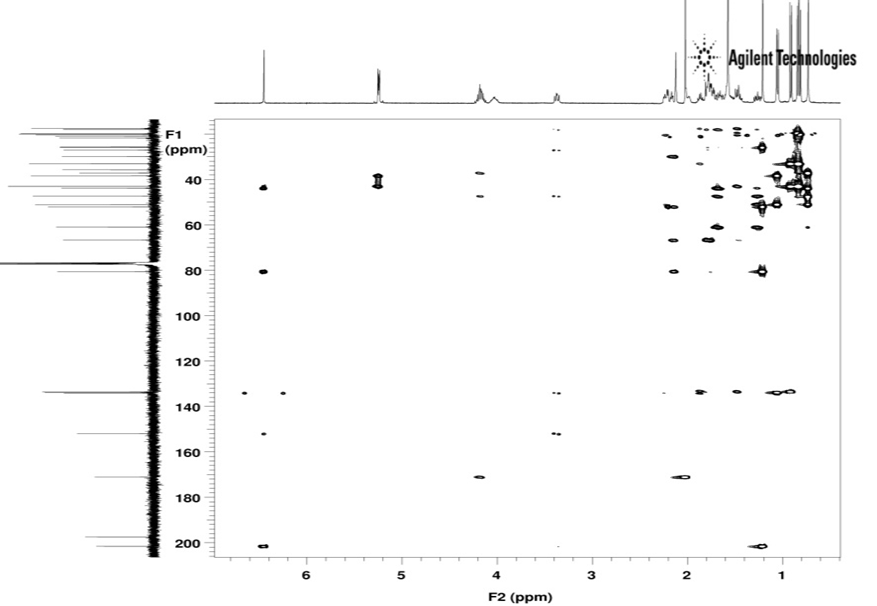


**Figure S6.** gHMBC spectrum (400 MHz) of compound **1** in CDCl_3_.


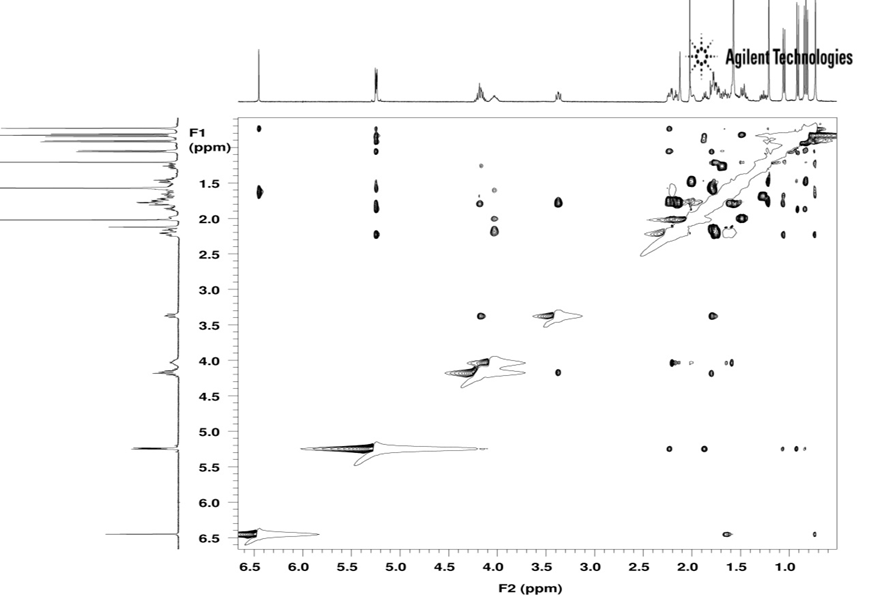


**Figure S7.** NOESY spectrum (400 MHz) of compound **1** in CDCl_3_.


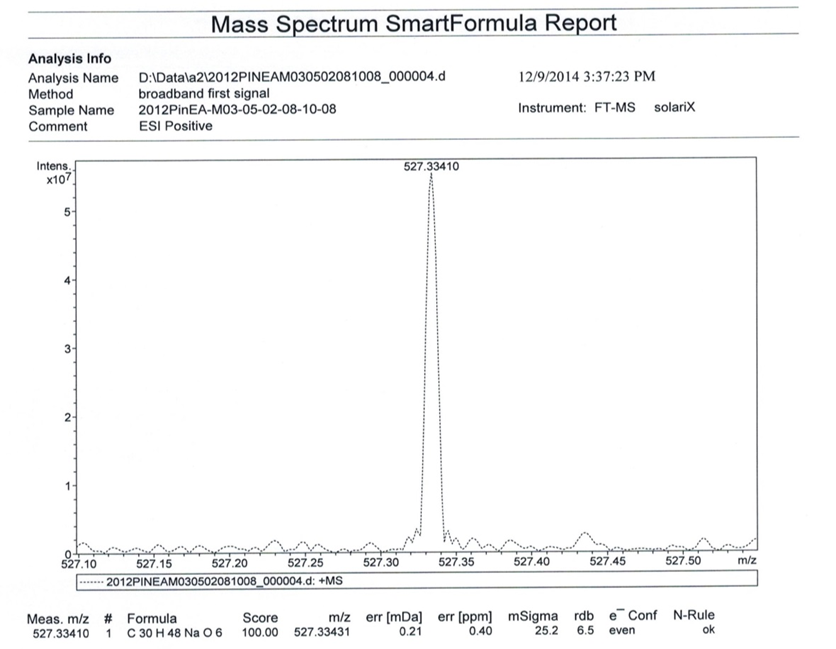


**Figure S8.** HRESIMS spectrum of compound **2**.


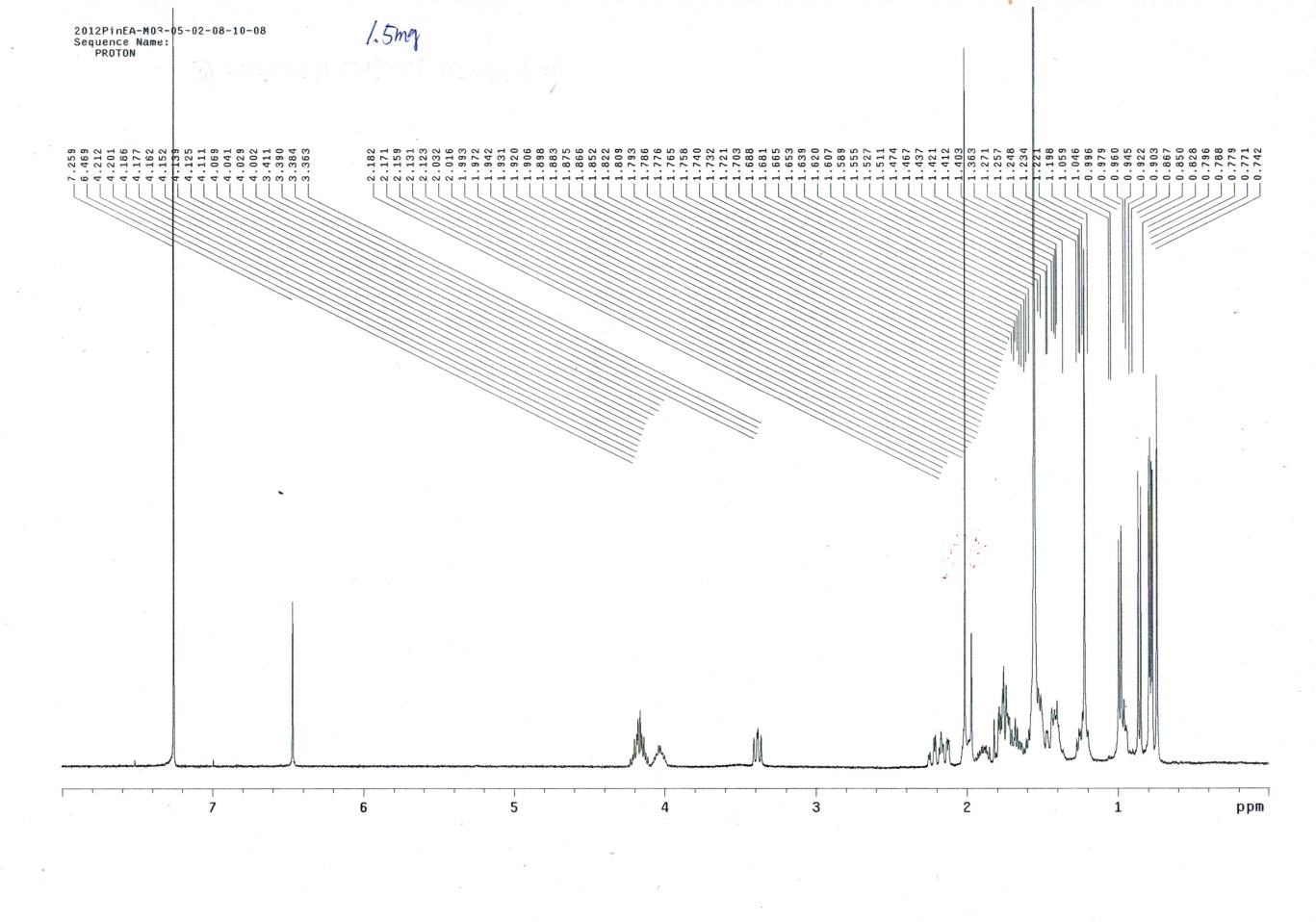


**Figure S9.** ^1^H NMR spectrum (400 MHz) of compound **2** in CDCl3.


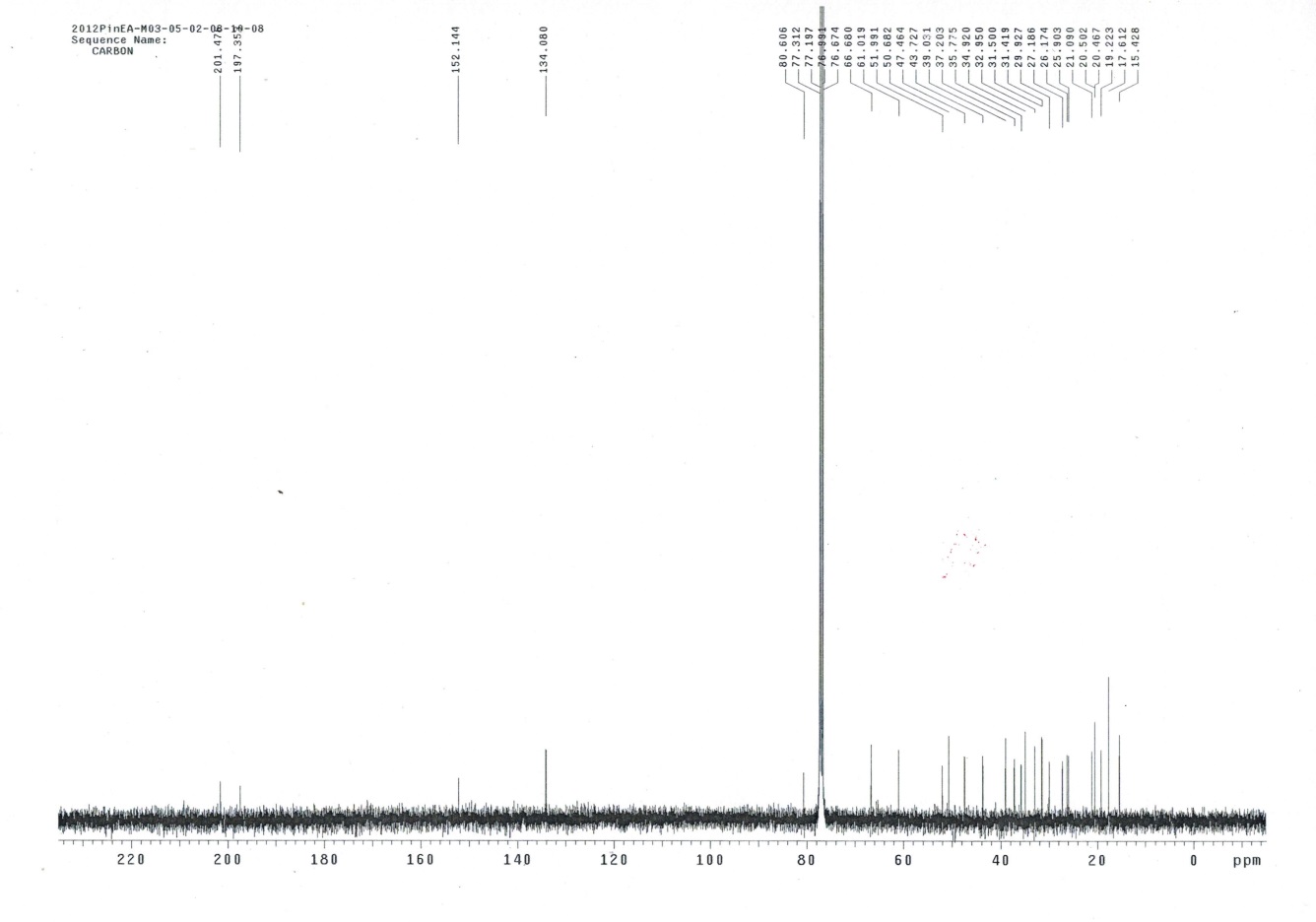


**Figure S10.** ^13^C NMR spectrum (100 MHz) of compound **2** in CDCl_3_.


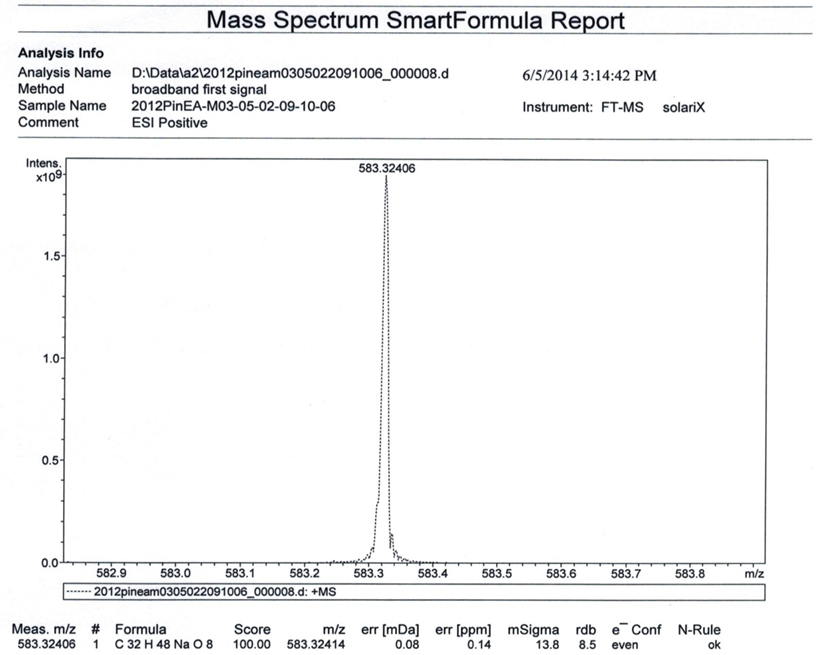


**Figure S11.** HRESIMS spectrum of compound **3**.


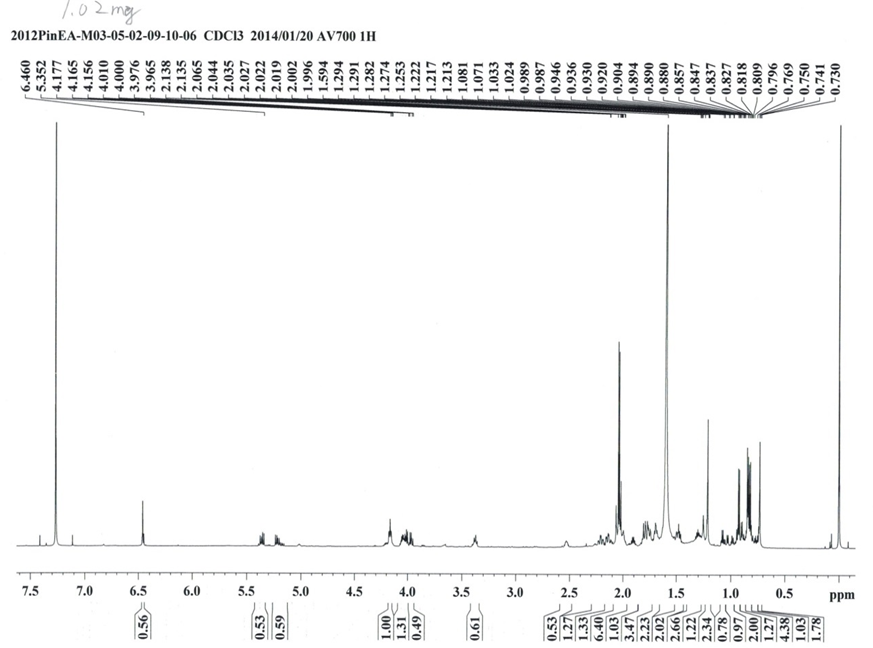


**Figure S12.** ^1^H NMR spectrum (700 MHz) of compound **3** in CDCl_3_.


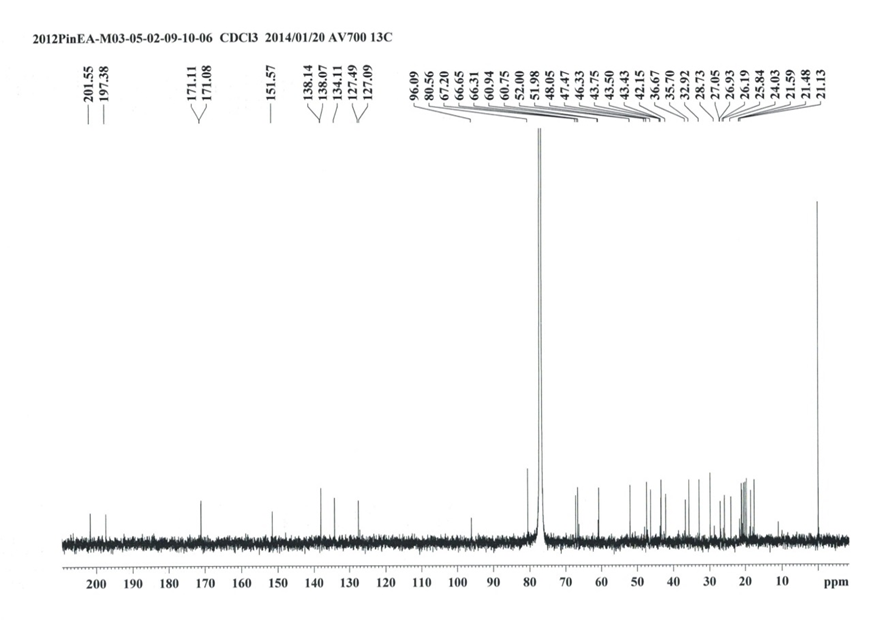


**Figure S13.** ^13^C NMR spectrum (175 MHz) of compound **3** in CDCl_3_.


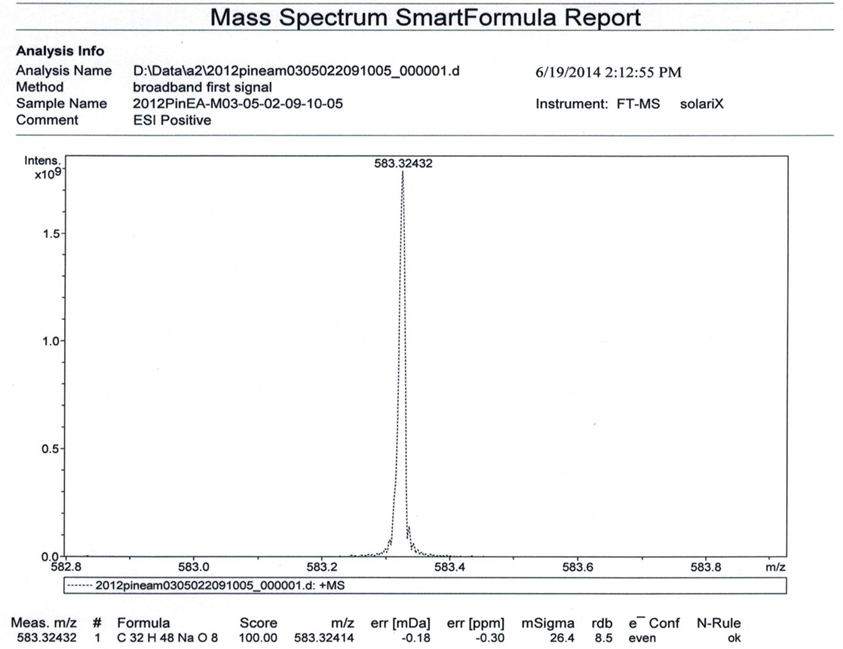


**Figure S14.** HRESIMS spectrum of compound **4**.


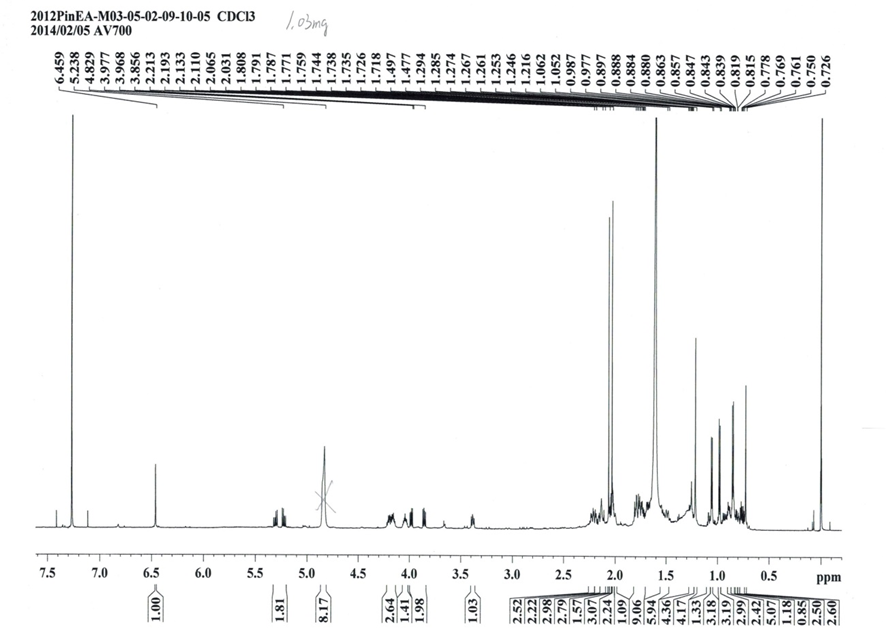


**Figure S15.** ^1^H NMR spectrum (700 MHz) of compound **4** in CDCl_3_.


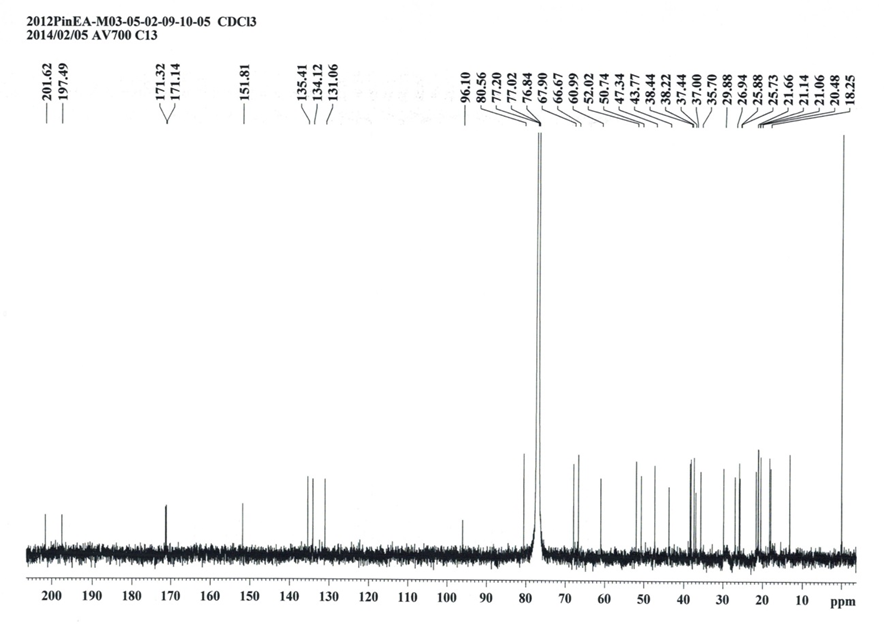


**Figure S16.** ^13^C NMR spectrum (175 MHz) of compound **4** in CDCl_3_.


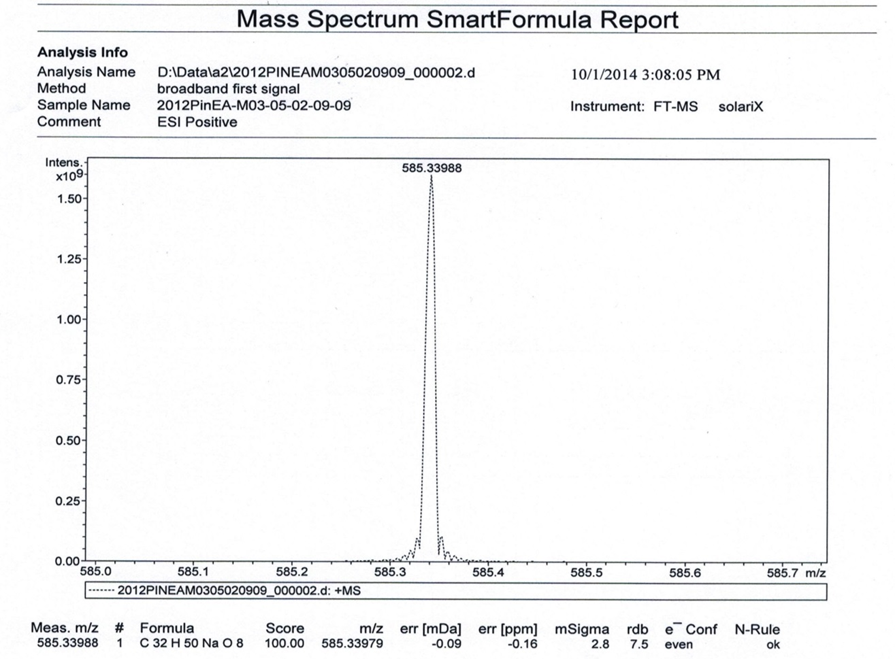


**Figure S17.** HRESIMS spectrum of compound **5**.


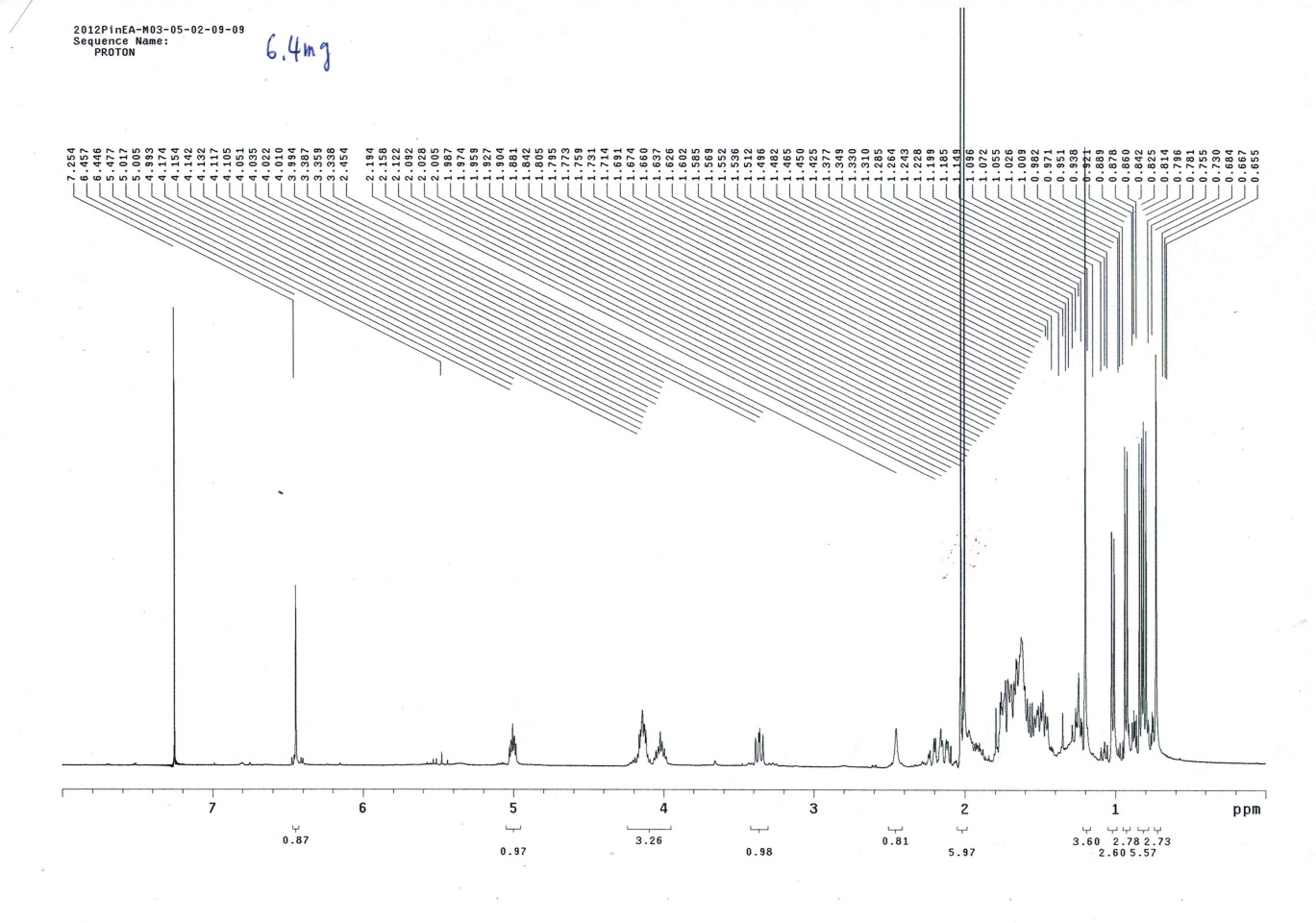


**Figure S18.** ^1^H NMR spectrum (400 MHz) of compound **5** in CDCl_3_.


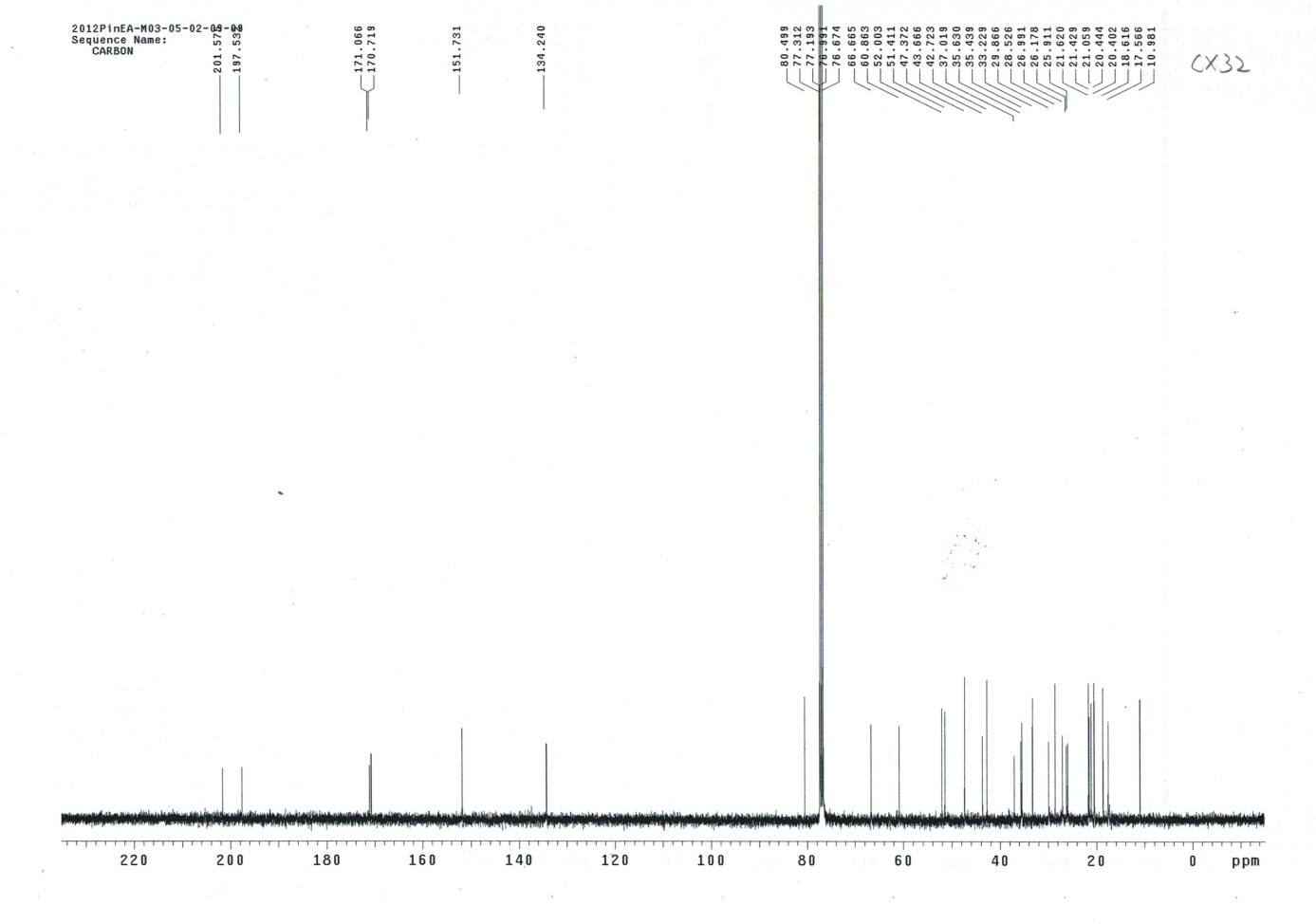


**Figure S19.** ^13^C NMR spectrum (100 MHz) of compound **5** in CDCl_3_.


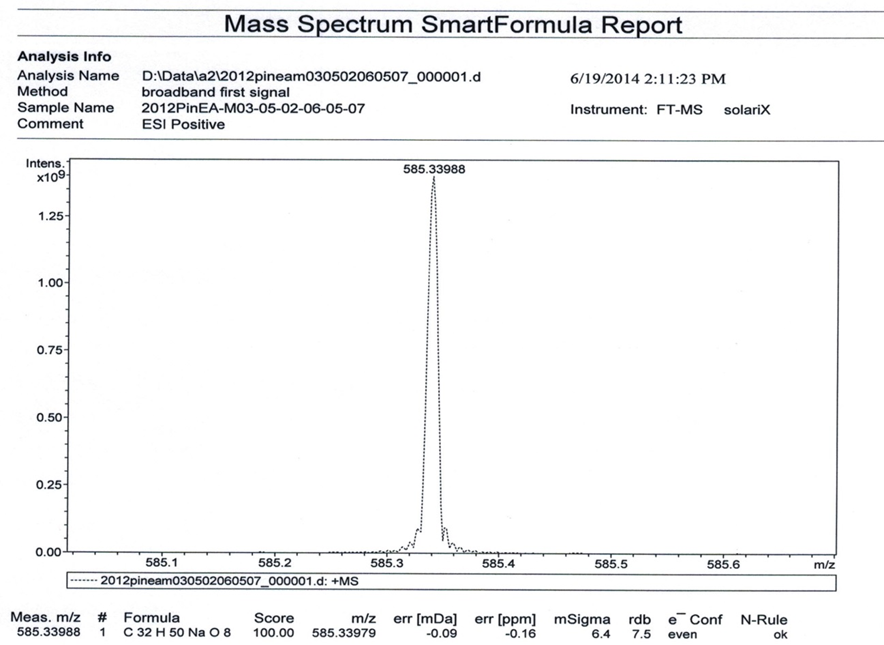


**Figure S20.** HRESIMS spectrum of compound **6**.


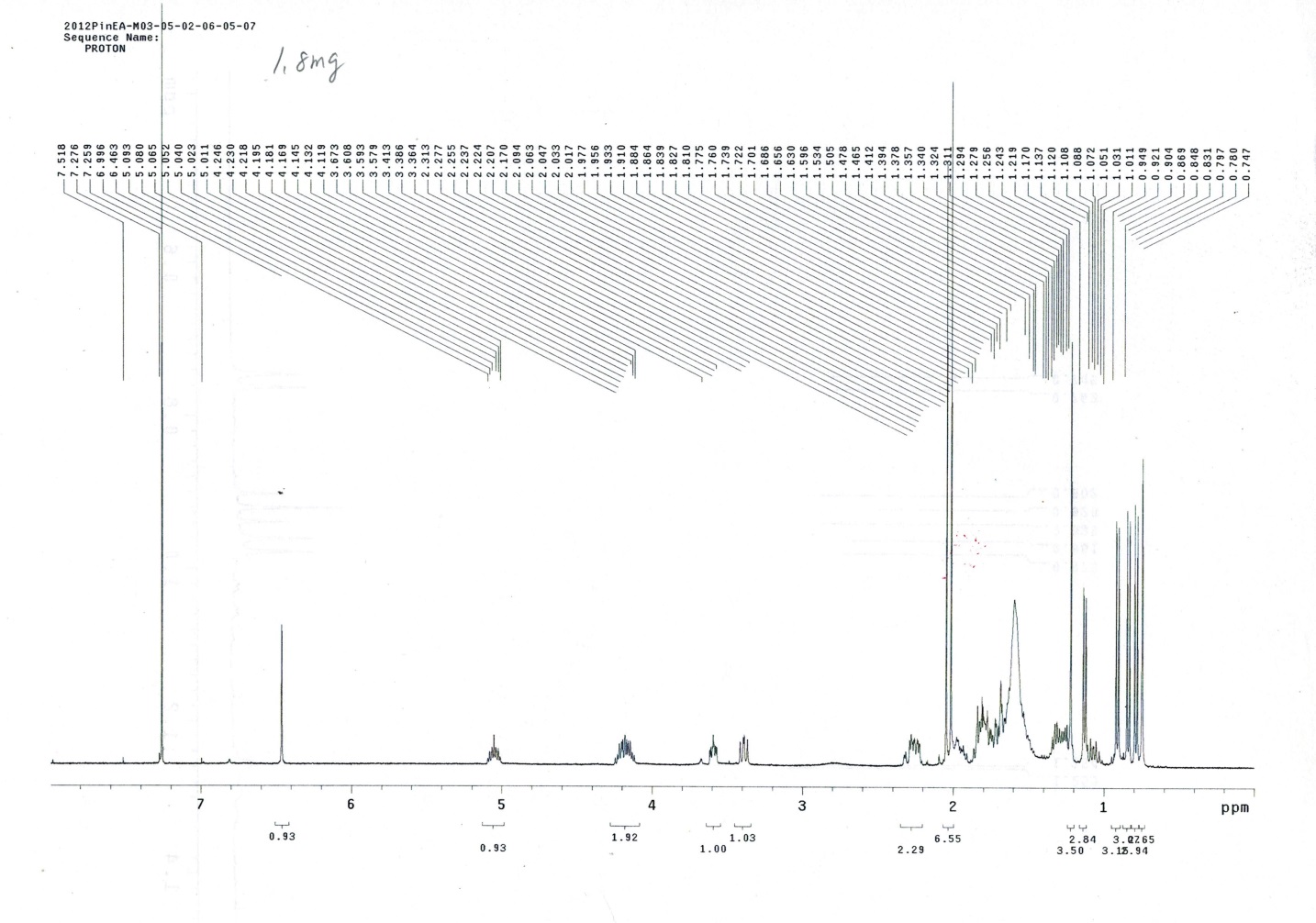


**Figure S21.** ^1^H NMR spectrum (400 MHz) of compound **6** in CDCl_3_.


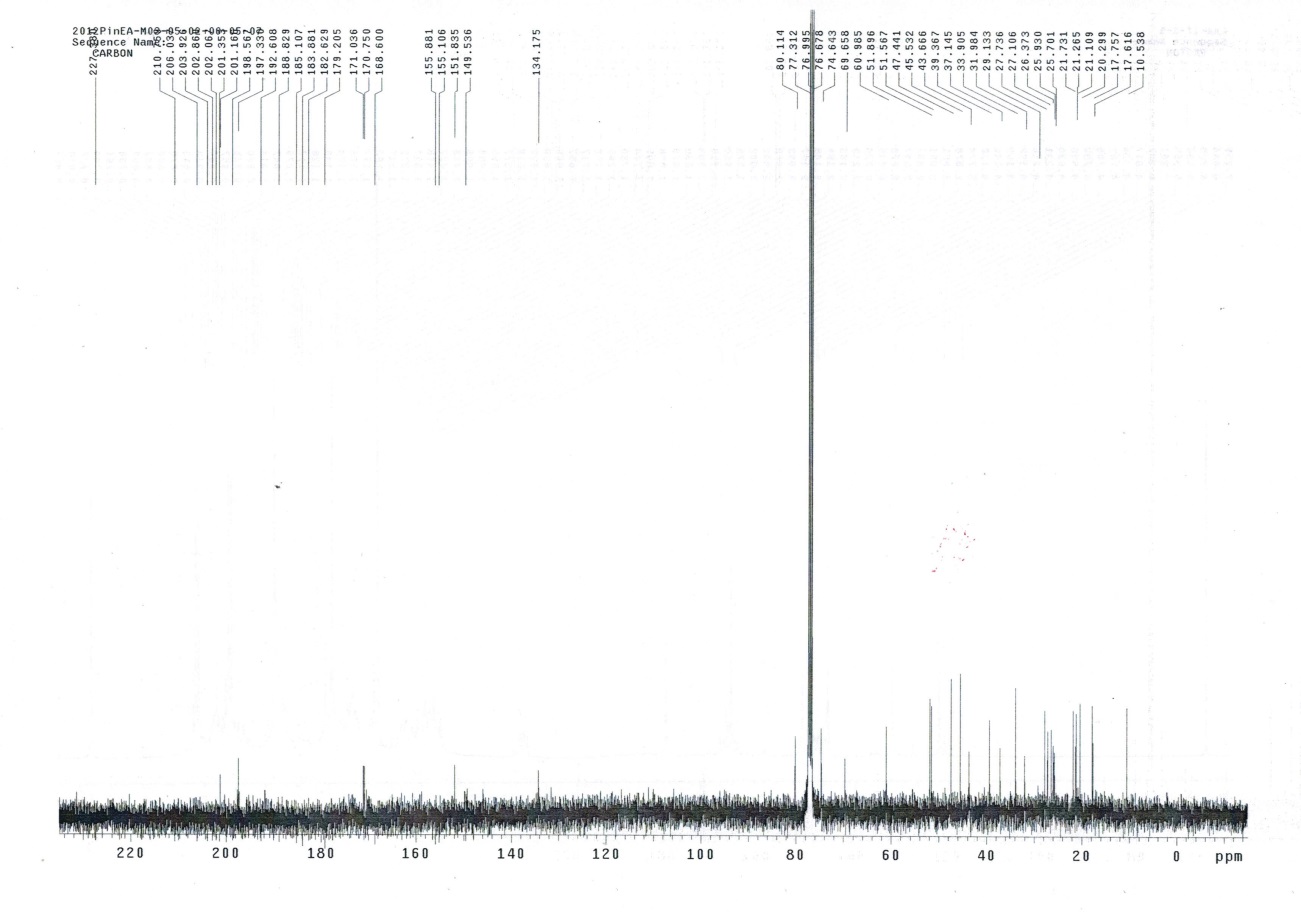


**Figure S22.** ^13^C NMR spectrum (100 MHz) of compound **6** in CDCl_3_.


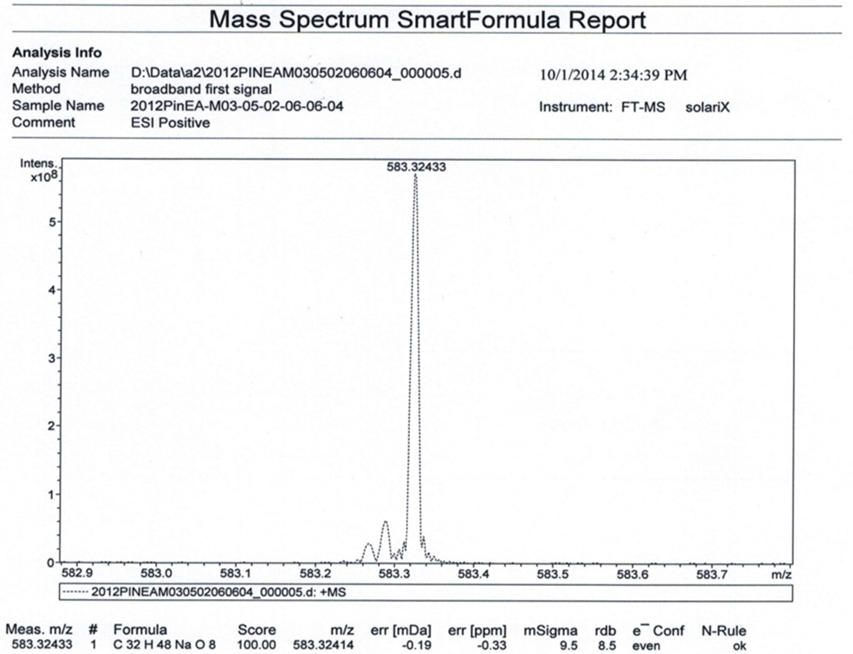


**Figure S23.** HRESIMS spectrum of compound **7**.


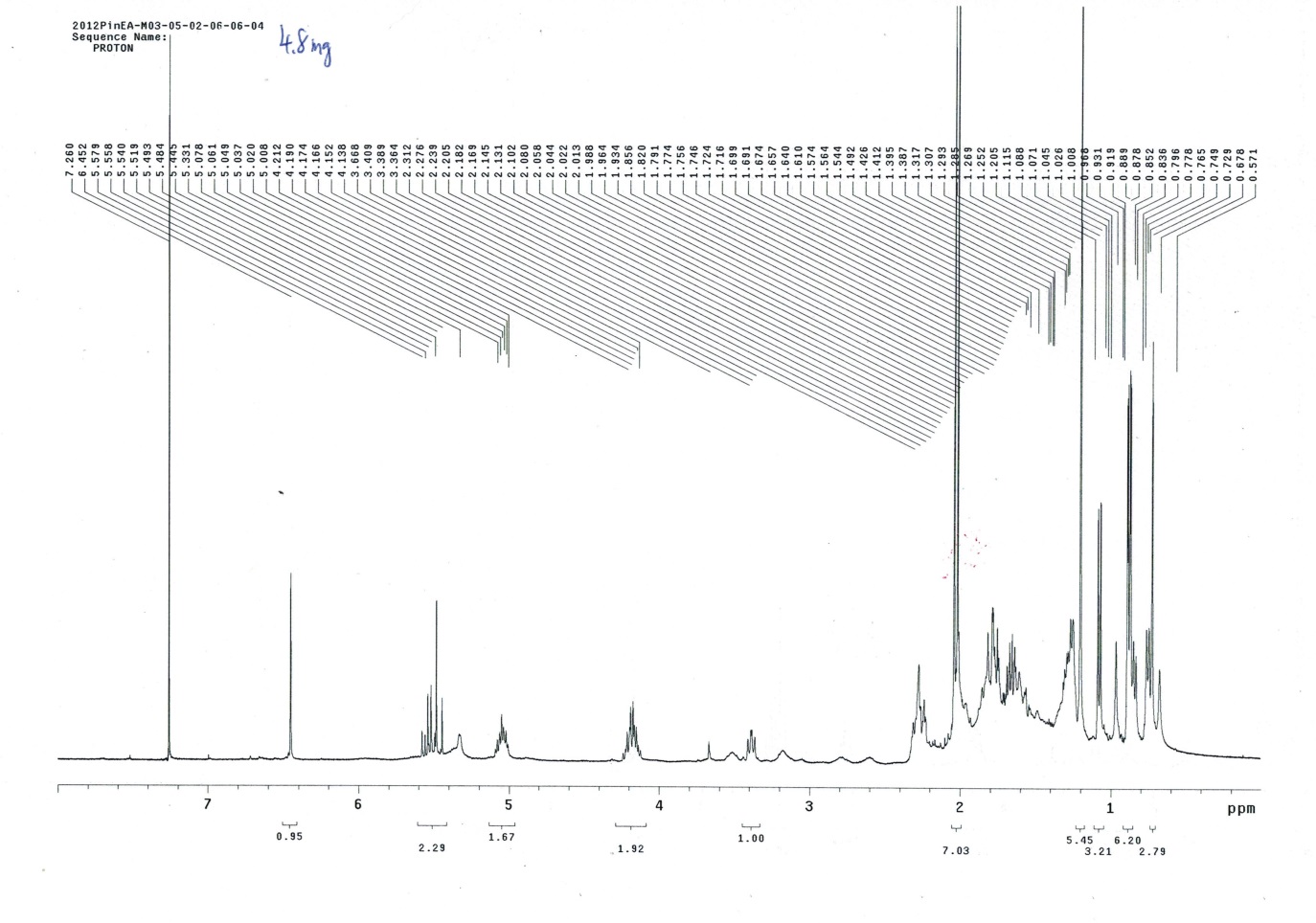


**Figure S24.** ^1^H NMR spectrum (400 MHz) of compound **7** in CDCl_3_.


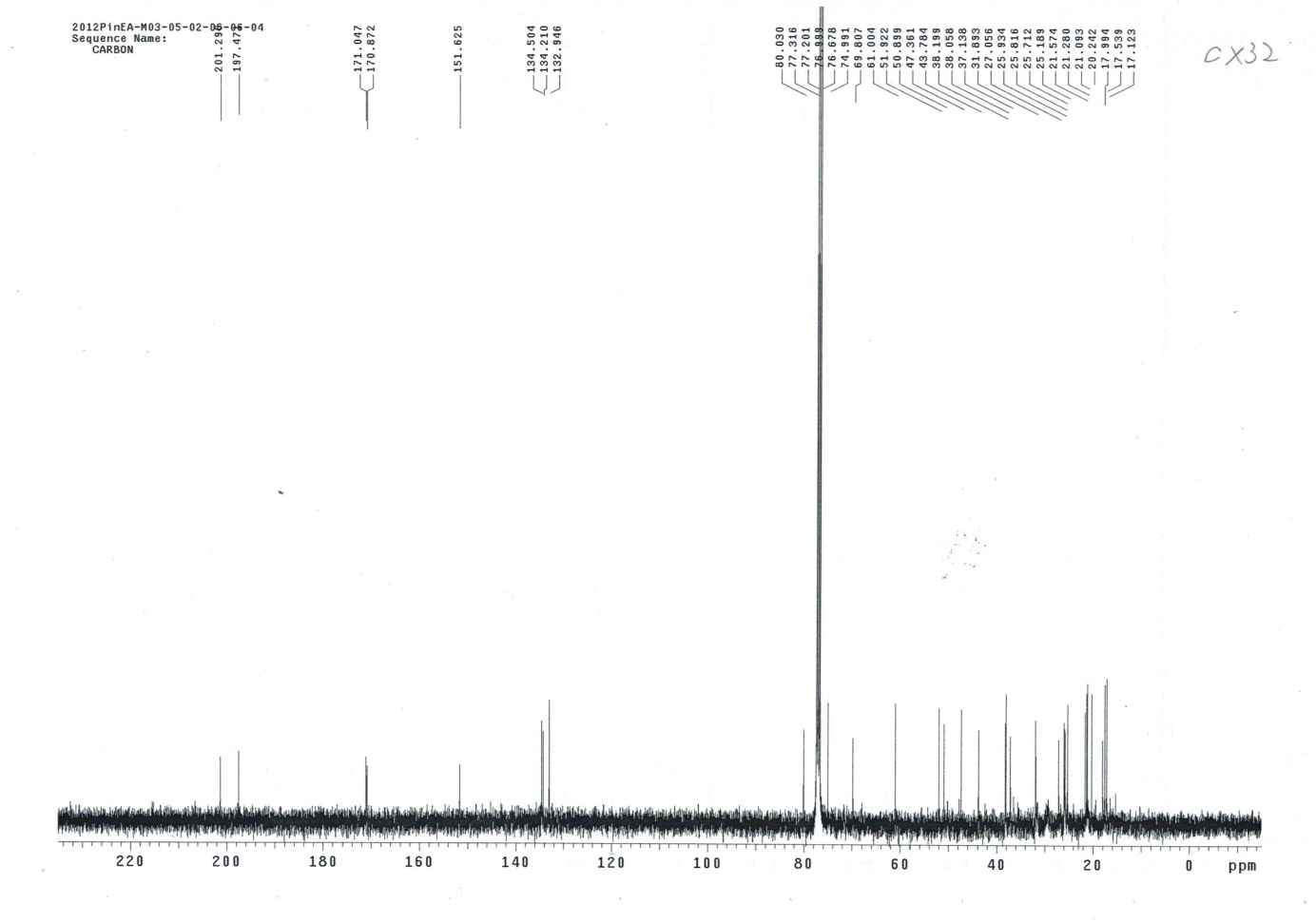


**Figure S25.** ^13^C NMR spectrum (100 MHz) of compound **7** in CDCl_3_.
